# Molecular-Scale Imaging Enables Direct Visualization of Molecular Defects and Chain Structure of Conjugated Polymers

**DOI:** 10.1021/acsnano.3c10842

**Published:** 2024-04-23

**Authors:** Stefania Moro, Simon E.F. Spencer, Daniel W. Lester, Fritz Nübling, Michael Sommer, Giovanni Costantini

**Affiliations:** † School of Chemistry, University of Birmingham, Birmingham B15 2TT, U.K.; ‡ Department of Chemistry, University of Warwick, Coventry CV4 7AL, U.K.; § Department of Statistics, University of Warwick, Coventry CV4 7AL, U.K.; ∥ Polymer Characterisation Research Technology Platform, University of Warwick, Coventry CV4 7AL, U.K.; ⊥ Institute for Macromolecular Chemistry, University of Freiburg, Freiburg 79104, Germany; # Institute for Chemistry, Chemnitz University of Technology, Chemnitz 09111, Germany; ∇ Center for Materials, Architectures and Integration of Nanomembranes (MAIN), Chemnitz University of Technology, Chemnitz 09126, Germany

**Keywords:** conjugated polymers, scanning tunnelling microscopy, homocoupling, mass distribution, sequencing

## Abstract

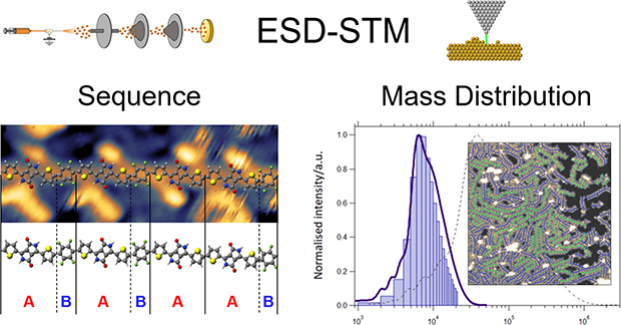

Conjugated polymers have become materials of choice for applications ranging from flexible optoelectronics to neuromorphic computing, but their polydispersity and tendency to aggregate pose severe challenges to their precise characterization. Here, the combination of vacuum electrospray deposition (ESD) with scanning tunneling microscopy (STM) is used to acquire, within the same experiment, assembly patterns, full mass distributions, exact sequencing, and quantification of polymerization defects. In a first step, the ESD-STM results are successfully benchmarked against NMR for low molecular mass polymers, where this technique is still applicable. Then, it is shown that ESD-STM is capable of reaching beyond its limits by characterizing, with the same accuracy, samples that are inaccessible to NMR. Finally, a recalibration procedure is proposed for size exclusion chromatography (SEC) mass distributions, using ESD-STM results as a reference. The distinctiveness of the molecular-scale information obtained by ESD-STM highlights its role as a crucial technique for the characterization of conjugated polymers.

## Introduction

Semiconducting conjugated polymers are attracting increasing attention in the broad field of organic electronics, as they offer tunable chemical structures, low-cost production, robust mechanical properties, and high charge mobility in applications. Their use ranges from organic photovoltaics (OPV)^1−3^ to organic field effect transistors (OFETs)^4,5^ and from organic thermoelectrics (OTEs)^6^ to (bio)sensors based on organic electrochemical transistors (OECTs).^7,8^ One of the major strengths of conjugated polymers when compared to inorganic semiconductors is the extreme variety of design pathways that can be followed to achieve, improve, or fine-tune their performance for specific applications. Typically, parameters that can be engineered to alter the performance of conjugated polymers in devices are (i) electronic structure of the materials, for example, by tuning the energy levels of the frontier orbitals and (ii) microstructural characteristics, such as backbone planarity, chain length, and chain–chain interactions resulting in ordered supramolecular architectures and eventually semicrystalline morphologies. Often, these properties are mutually dependent and thus a deep understanding of both electronic and structural characteristics and of their interdependence is necessary, not only to explain different behaviors and performances in devices, but also to improve the design of new materials.^9−11^

Precise determination of chain length and chain length distribution is central to understanding structure–function relationships of these polymers in devices and thus constitutes a fundamental milestone in the pathway to producing high-performance materials.^12−16^ However, the analytical tools that are currently available and routinely used for investigations of the structural and chemical properties of these materials still struggle to consistently provide exact information. Size exclusion chromatography (SEC) is typically used for average mass (*M*_n_, *M*_w_, *M*_*z*_) determination but is known to suffer from systematic overestimation due to the calibration being done with polystyrene, a material with drastically different hydrodynamic properties from conjugated polymers. The issue of relative calibration and overestimation can be addressed with more elaborate detection such as viscosimetry, static light scattering, or field flow fractionation that enable universal calibration, as has been shown for individual examples.^17−20^ Moreover, aggregation is often present, enhancing the problem of overestimated molar masses. At higher temperature, aggregates can be dissolved depending on the system and, thus, high-temperature SEC is a viable option.^21,22^ By combining SEC with other techniques such as nuclear magnetic resonance (NMR) and matrix-assisted laser desorption/ionization time-of-flight mass spectrometry (MALDI-ToF MS), characterization of molecular weight of conjugated polymers with better accuracy has been achieved.^23−25^ However, all of these combinations and techniques suffer from drawbacks, being limited information, tedious handling, extensive costs, or a combination of those. For example, while MALDI-ToF MS delivers distributions, absolute average masses and information on end groups,^23^ this method tends to underestimate molecular weight and dispersity.^26,27^ This is especially problematic for conjugated polymer samples made by polycondensation and having broad mass distributions.^28^ Finally, NMR can be reliably used for average mass determination and for sequencing of conjugated polymers, but this method is limited to lower molar masses.^24,27,29^

A further essential aspect for establishing reliable structure–function relationships in conjugated polymers is the identification and quantification of defects in the polymer sequence caused by errors in the polymerization process. This is true for cross-coupling techniques such as direct arylation polycondensation (DArP),^30−33^ that use shorter reaction sequences and less toxic reagents, aiming at greener reaction pathways. However, it is interesting to note that defects such as homocoupling reactions have been reported for copolymers made also by other cross-coupling techniques, including Suzuki and Stille polycondensation,^34−39^ suggesting that a large fraction of materials investigated so far are defective. A number of possibilities exists for defect formation in cross-coupling techniques, including unselective C–H activation, homocoupling (hc) reactions of equal monomers, or reactions with solvent, ligand or other components of the catalytic system.^40−42^ Among these possibilities for deviations from the ideal catalytic cycle, hc reactions are the most prevalent and have the potential to significantly influence optoelectronic properties. The extent to which this happens is, however, strongly system-dependent. For example, for carbazole-based PCDTBT and diketopyrrolopyrrole (DPP) copolymers used as hole transporters in polymer/fullerene solar cells, carbazole, and DPP hc are detrimental to device performance.^35,43,44^ Similarly, hc defects have shown to negatively affect fullerene intercalation and charge-transfer absorption in pBTTT/fullerene blends for photovoltaic applications.^37^ However, there are also indications that electron mobility of DPP copolymers used in *n*-channel transistors is not strongly influenced by hc.^45^ Identification and quantification of such defects have been attempted with MALDI-MS and NMR but this approach is severely limited. In this case, the main issue with MALDI-MS is the fact that this technique is not inherently quantitative while, despite major recent progress, the intrinsically rather low sensitivity of NMR and the need for reference models render defect identification and quantification in the few percent range extremely cumbersome.^34,43,46,47^

Here, we demonstrate that the combination of electrospray deposition (ESD) of conjugated macromolecules with ultrahigh vacuum (UHV) high-resolution scanning tunnelling microscopy (STM) imaging is a solution to the lack of reliable and multifunctional analytical tools to investigate conjugated polymers.^37,48−54^ In particular, we demonstrate how high-resolution STM imaging of conjugated polymers, combined with advanced statistical approaches, can be used to simultaneously gather precise information on: (i) intermolecular interaction patterns; (ii) mass distribution; and (iii) direct sequencing with in-depth information on their chemical structure and molecular defects. In particular, we first benchmark the validity of the ESD-STM analytical approach against results from ^1^H NMR spectroscopy for low number-average molecular mass (*M*_n_) polymers that have been specifically synthesized as model systems exhibiting sufficient NMR baseline resolution. For these materials, the ESD-STM data show excellent quantitative agreement with the NMR results of the *M*_n_ values and the type and average frequency of homocoupling defects. In a second step, we show that ESD-STM allows for this information to be obtained, with the same level of accuracy, also for polymers for which a quantitative analysis of the ^1^H NMR spectra is hampered by polymer aggregation. Furthermore, we propose a recalibration procedure for SEC mass distributions, using ESD-STM results as a reference. Our work thus demonstrates that, when applied to conjugated polymers, the ESD-STM technique is capable of reaching beyond the limits of traditional analytical techniques in terms of materials that can be measured, level of molecular-scale details that can be achieved and, sometimes, even the type of information that can be obtained.

## Results and Discussion

**Figure 1:**
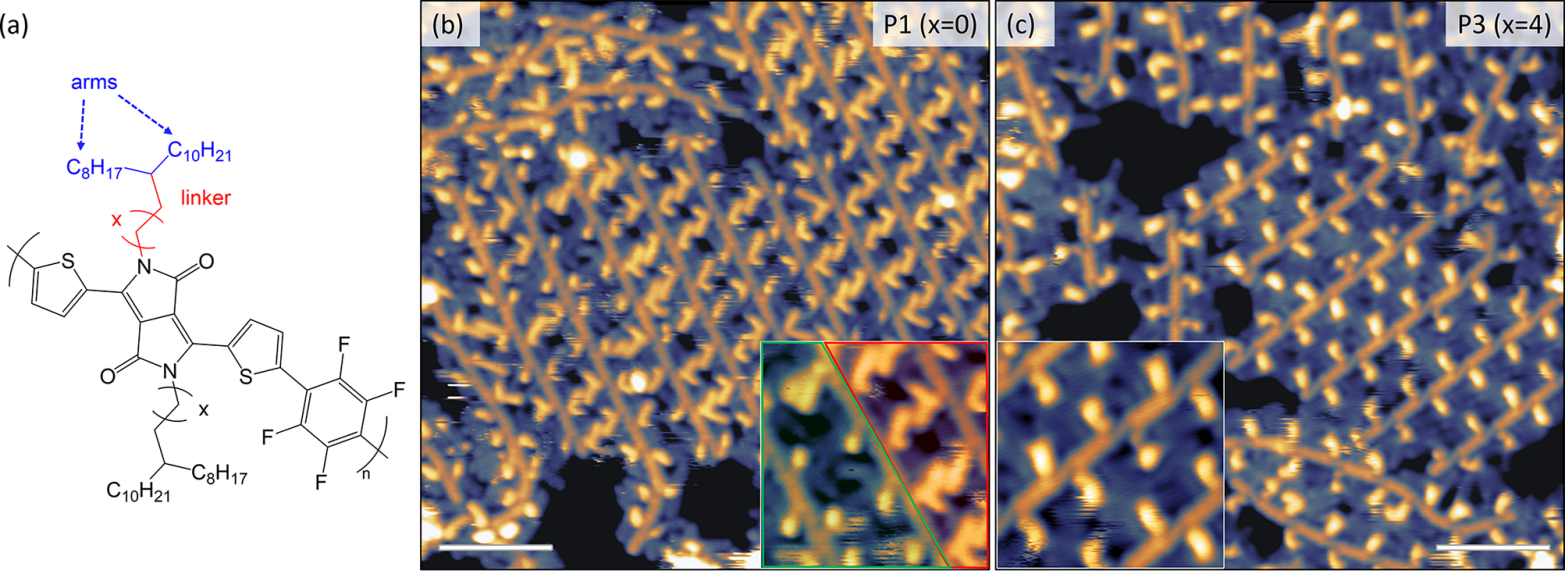
(a) Chemical structure of PThDPPThF4. The colored parts of the side chain indicate the linear linker of different length (red) and two asymmetric branched arms (blue). High-resolution STM images are shown in (b) and (c) for P1 and P3, respectively. The inset in (b) displays an example of the ordered parts of the assembly (top-right corner, in red) and a case where side chains are not arranged into a regular pattern (bottom-left corner, in green). The inset in (c) shows an ordered area of P3. The scale bar in (b) and (c) is 4 nm, and the lateral size of the insets is 5 nm. STM images were acquired in constant current mode with tunneling parameters: (b) 1.4 V, 70 pA; (c) 1.3 V, 90 pA.

The materials studied in this work are based on widely employed DPP motifs that are rigid and strong absorbers with high mobility, and are of great relevance for photovoltaic and charge transport applications.^1,2,55,56^ Specifically, the investigated PThDPPThF4 copolymers comprise alternating dithienyldiketopyrrolopyrrole (ThDPPTh) and tetrafluorobenzene (F4) units prepared by DArP. The first polymer, P1, (Figure 1a, *x* = 0) was chosen for benchmarking the experiment since, despite its strong aggregation in solution, it shows sufficient solubility to be well-characterized by ^1^H NMR.^34,45,57^ Moreover, PThDPPThF4 has been extensively studied by density functional theory (DFT) calculations and grazing incidence wide-angle X-ray scattering (GIWAXS) and shows high mobility.^57^ P2 has the same structure as P1 but was synthesized under different conditions with the aim of having a different hc defect content. P3, instead, shares the same backbone structure as P1 and P2 but its side chain branching point is further away from the backbone (Figure 1a, *x* = 4). While this improves the polymer assembly capability and thus produces more ordered thin films for device applications,^58−60^ the stronger aggregation of P3 makes ^1^H NMR spectra with a baseline resolution elusive even at elevated temperature. Details of synthetic procedures are given in the Supporting Information (SI). In the following, the linear aliphatic side chain between the aromatic backbone and the branching point will be referred to as a linker (red in Figure 1a), while the two branches will be called arms (blue in Figure 1a).

As mentioned, among the factors influencing the efficiency of conjugated polymers in devices, a major role is played by their chain length (often indicated also as molecular weight).^13,15,16^ With the aim of determining this, all three polymers have been preliminarily characterized by standard analytical methodologies. P1 and P2 yield ^1^H NMR spectra with sufficient resolution such that assignments of end groups and determination of the number-average molar masses (*M*_n_) is feasible (see Figures S7 and S8 and Table 1). In contrast to P1 and P2, the extended side chains of P3 and the subsequent increased aggregation of the backbones cause significant broadening of the peaks in the ^1^H NMR spectra (see Figure S9). In this case, it is therefore not possible to assign and quantify end group intensities for *M*_n_ determination (see Table 1).

**Table 1:** Comparison of Average Mass Values (DP_n_, *M*_n_, *M*_w_) for P1, P2, and P3, Obtained by Different Experimental or Statistical Approaches^a^

		P1	P2	P3
DP_n_	STM	8.7 ± 0.1	7.7 ± 0.1	7.6 ± 0.1
	KM	10.2 ± 0.2	9.5 ± 0.2	9.2 ± 0.2
	parametric fit	9.9 ± 0.2	9.3 ± 0.3	9.0 ± 0.2
	^1^H NMR	9.3 ± 1.0	7.5 ± 3.0	--
	SEC	42.3	32.2	67.7
*M*_n_/kDa	STM	8.5 ± 0.1	7.5 ± 0.1	8.3 ± 0.1
	KM	10.0 ± 0.2	9.3 ± 0.2	10.0 ± 0.2
	parametric fit	9.7 ± 0.2	9.1 ± 0.2	9.8 ± 0.2
	^1^H NMR	9.4 ± 1.0	7.5 ± 3.0	--
	SEC	41.5	31.6	74.0
*M*_w_/kDa	STM	9.6 ± 0.3	8.9 ± 0.3	9.4 ± 0.3
	KM	11.4 ± 0.5	11.1 ± 0.5	11.9 ± 0.7
	parametric fit	11.4 ± 0.5	11.5 ± 0.6	12.0 ± 0.5
	SEC	88.5	103.4	358.9

a ^1^H NMR spectra were measured in C_2_D_2_Cl_4_ at 120 °C, SEC was measured in chloroform at 30 °C.

All three polymers were also measured by ESD-STM with the aim of determining their molecular weights. The samples were prepared by electrospray deposition of the polymers under identical conditions onto an atomically clean Au(111) substrate kept at room temperature in a vacuum (see the SI for further details). The deposition parameters were chosen so as to form a monolayer of polymer chains. The self-assembled polymers were then imaged by UHV-STM at 77K. In the STM images, all three polymers are seen to adsorb face on onto the surface and show a tendency to form compact islands characterized by interacting polymers (Figure S4). In closely packed regions (see Figure 1b,c for P1 and P3 and Figure S5a for P2), individual polymer chains can be identified in the images through extended, straight and continuous features, corresponding to their backbones.

The interbackbone distance (*i.e*., the 2D equivalent of the lamellar spacing in 3D films) is (2.0 ± 0.1) nm for P1 and P2 and (2.5 ± 0.2) nm for P3. For P1 and P2, this is in excellent agreement with the value of 2.04 nm (2.11 after annealing) obtained for the lamellar spacing of thin films of the same polymers by GIWAXS.^57^ For all three polymers, these values are consistent with the interdigitation of their side chains.

The branched side chains are seen in the STM images as shorter, bright, and, in the case of P1 and P2, mostly angled features that flank the backbones. As shown in Figure 1b,c, different assembly patterns and degree of regularity can be found for *x* = 0 (P1, P2) and *x* = 4 (P3). For P1 and P2, in the more regular areas of the assembly, the branched side chains appear as bright L-shaped objects with arms of different lengths (red part of the inset in Figure 1b). In disordered or defective areas of the assembly (the green part of the inset in Figure 1b), the only part of the side chains that appears bright in the STM images corresponds to the carbon located nearest to the lactam-N of ThDPPTh, while the two arms appear darker and form rather irregular patterns. This is due to the presence of sp^3^ carbon atoms and the associated tetrahedral angles.^51^ The side chains of P3 instead appear as bright segments protruding from the backbone at a typical angle of 60 ± 1° followed by a darker pair of segments that form an overall uniform assembly (inset in Figure 1c).

**Figure 2:**
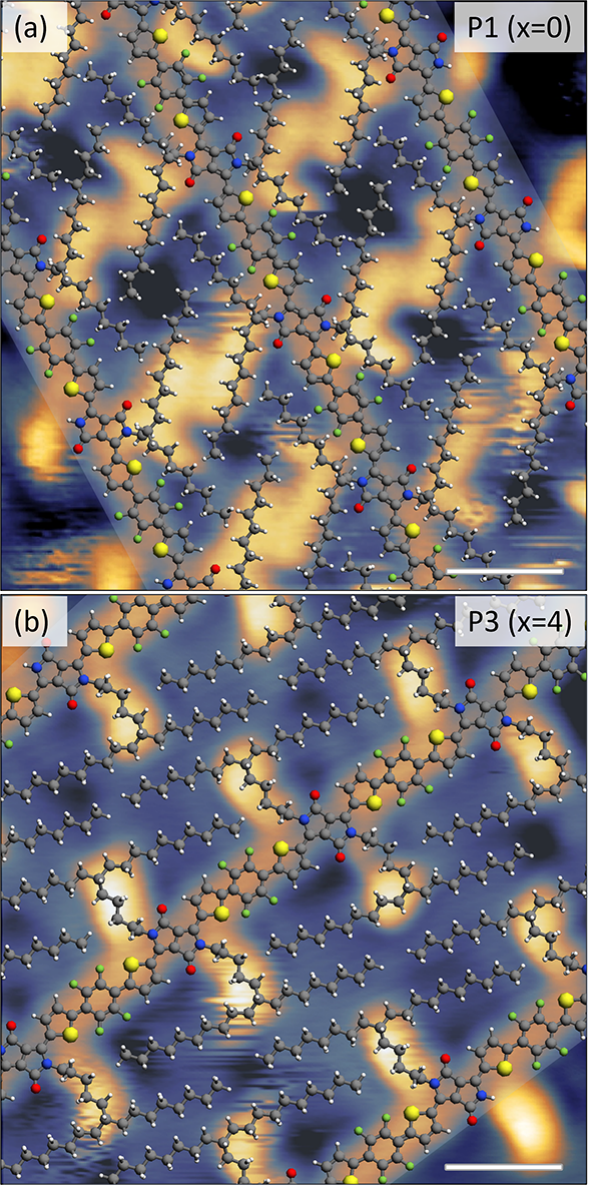
In (a) and (b), geometry-optimized molecular models superposed onto high-resolution STM images are shown for P1 and P3, respectively. This modeling allows one to identify the details of the backbone sequence and of the polymer assembly, including complex side chain interdigitation patterns. All scale bars correspond to 1 nm. The STM images were acquired in constant current mode, with tunnelling parameters (a) 1.4 V, 70 pA; (b) 1.3 V, 140 pA.

In order to exactly identify the regular patterns formed by P1 and P3, geometry-optimized molecular models were built in the Avogadro molecular editor (using the MMFF94 force field) and fitted to the STM images. For the *x* = 0 polymers (P1 and P2), the areas of regular side chain packing are well represented by models in which the arms form a 120° angle with the linker and among themselves, as shown in Figure 2a and Figure S5a for P1 and P2, respectively. The shorter arms (C_8_H_17_) tend to orient almost parallel to the backbones, possibly interacting with the F atoms of the F4 units. The longer arms (C_10_H_21_) interdigitate with other longer arms from adjacent polymers, most probably through van der Waals (vdW) forces. For P3, instead, the best fitting model shows the two arms orienting along a single line, with the shorter and longer arms forming angles of 60° and 120° with the linker, respectively (Figure 2b). This conformation allows very favorable vdW interactions and thus dense packing of side chains from neighboring polymers. Overall, the proposed fits represent all of the features visible in the STM images. Details of the fitting procedure can be found in Section 3 of the SI.

The optimal fit of the molecular models to the STM images grants access to the full conformations of the backbones, and the prevalent configuration adopted by the polymer backbones can be inferred. For all three polymers, the DPP units show a N,S-*syn* orientation with respect to the neighboring thiophene rings, with an *anti* configuration of the thiophenes flanking a single F4 unit. This is in excellent agreement with solid state NMR results and DFT simulations on PThDPPThF4 (*x* = 0) polymers, that identified this as a minimum energy configuration stabilized by an intramolecular conformational lock between the hydrogen atom in the thiophene units and the carbonyl oxygen of the DPP core.^57,61^ By ESD-STM, however, it is also possible to establish the presence of other, minority configurations that are not detectable by standard averaging characterization techniques. In fact, occasional instances where the DPP units are in N,S-*anti* orientation with respect to one of the flanking Th rings were also observed (see Figure S6a for an example).

Even though the presence of the gold substrate might have an influence on the intermolecular interactions, we expect that several features of the assembly observed in the 2D monolayers will characterize also the packing of these polymers in 3D thin films, as already reported in other cases.^53^ A first confirmation of this is given by the excellent agreement between the lamellar spacings measured by GIWAXS and those determined through STM, and by the analogies of the backbone conformation as determined by STM and solid state NMR. However, we expect that this similarity also extends to other structural details of the molecular assembly, such as the interaction motifs between the side chains. Thus, we expect the patterns observed in the STM images (Figure 2) to be a good representation of those characterizing the side chain interaction in 3D thin films, which are typically challenging to determine by X-ray diffraction or solid state NMR.

Based on this detailed interpretation of the ESD-STM data, it becomes now possible to determine the polymer molecular weight through direct analysis of STM images by measuring the length of individual polymer chains. Even more directly, in high-resolution images, one can count just the number of repeat units. Unlike more traditional techniques such as viscosimetry, SEC, and NMR, polymer aggregation is not expected to represent a limitation for ESD-STM. Aggregation within the microdroplets produced during the ESD process is a known phenomenon that does not prevent the formation of the monolayers required for STM analysis. Single polymer strands are clearly distinguished in STM images of these assembled monolayers and are individually accounted for in the analysis. Moreover, since ESD is not a mass-selective deposition technique,^62^ the polymers measured by STM are expected to represent an unbiased sample of the original material. As a consequence, when this analysis is performed on a large enough set of data, the results can be taken as a representative description of the polymer mass distribution. This is in contrast to MALDI-MS, where shorter chains are detected with higher probability leading to underestimated molecular weight distributions.^26,63^ A further significant strength of ESD-STM over traditional techniques is that not only does it provide a description of the polymers’ mass through average quantities (such as the number-average molar mass, *M*_n_, the weight-average molar mass, *M*_w_, and the degree of polymerization, DP_n_) but it also gives the complete mass distribution of the material, thus allowing for a much more accurate description of the polymer population. In the following, we will first benchmark the ESD-STM results on the mass of the PThDPPThF4 polymers against the NMR-derived data for P1 and P2 and then present the mass distribution for P3, which cannot be obtained from NMR (*vide supra*).

**Figure 3:**
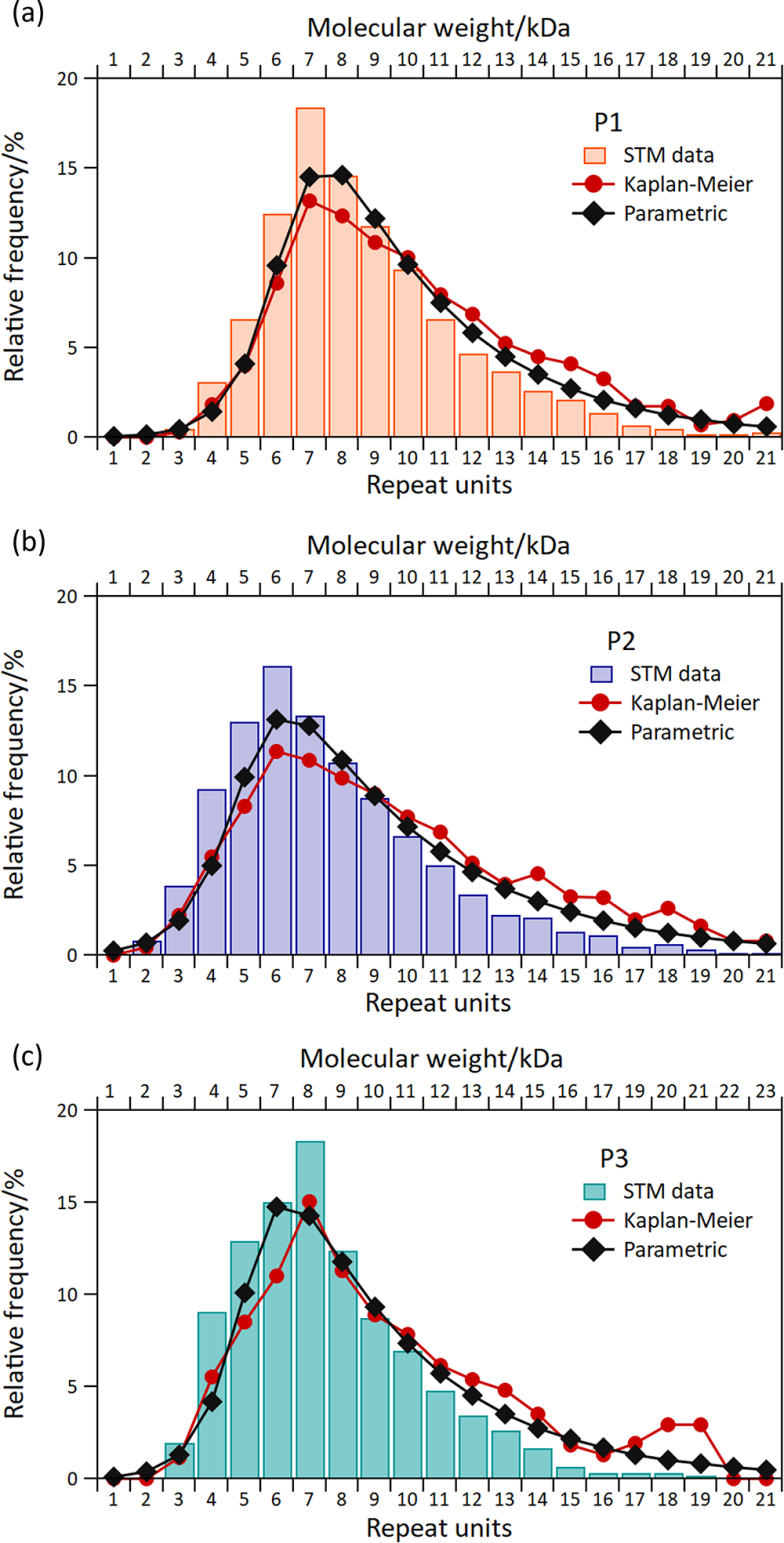
Length and mass distributions obtained from measuring a large number of polymer line-profiles from the STM images. Only polymers fully visible and entirely contained within the STM images were used for the bar histogram distributions (see Figure S11). (a) Distribution for P1, (b) for P2, and (c) for P3. Two statistical approaches from survival analysis have been implemented to correct the length underestimation caused by polymers either not fully visible or not entirely contained within the STM images: the Kaplan–Meier nonparametric distribution (red circles) and the parametric distribution (black diamonds). See the text for further details.

For the ESD-STM analysis, a large number of polymer lengths have been collected for all three molecules, and the experimental distributions are shown as bar histograms in Figure 3. The corresponding average values are reported in Table 1 and the details on the line-profile acquisition can be found in Section 5 of the SI. This approach has a limitation in the fact that only polymers that are fully distinguishable from the beginning to the end can be directly included in these distributions (see green lines in Figure S10b). Polymers that cross through the borders of the STM images and polymers whose line-profiles cannot be unambiguously distinguished in their entirety–for example because they cross another polymer or traverse a poorly resolved part of an image–are excluded from the initial length distributions (see blue lines in Figure S10b). Both of these cases tend to affect longer polymers more than shorter ones. Since almost half of the polymers present in an STM image pertain to such excluded categories, not accounting for their presence can produce a significant skewing of the final histograms toward smaller masses. We therefore tackled this issue by means of a statistical analysis based on the survival analysis approach.^64^

In essence, this method consists of combining the distribution of the fully visible polymers (green in Figure S10b) with the distribution of polymers that have been classified as “longer than” (blue in Figure S10b), based on their maximal unambiguously determined length. In particular, the latter distribution is used to “correct” the former by taking into account that the observed incomplete polymers must be a section of longer molecules. Among the different possibilities offered by survival analysis, we chose two complementary approaches for performing this correction: a nonparametric approach (with a Kaplan–Meier estimator) that makes no *a priori* assumption on the final mass distribution, and a parametric approach where a Flory–Schulz (geometric) mass distribution was assumed, according to what is expected from an ideal step-growth polymerization process. Moreover, in order to take into account the effects of the purification processes that cause the preferential removal of shorter polymers (e.g., Soxhlet extraction purification), the geometric Flory–Schulz distribution was multiplied by a logistic curve (see Section 6 of the SI).

The results of these two approaches are overlaid as red dots and black diamonds, respectively, on the histogram distributions directly derived from the STM images in Figure 3. It is clear that both corrections move the distributions toward higher masses, as expected. The same effect is visible in the evaluation of the average quantities *M*_n_, *M*_w_, and DP_n_, where the values obtained from the Kaplan–Meier and the parametric corrections are all larger than those obtained from the direct STM analysis (Table 1). The *M*_n_ values estimated with the two different statistical correction approaches only differ by about 2%, showing that as long as the intrinsic limitations of finite size STM images are taken into account, the specific approach used does not significantly influence the result. These corrected distributions, therefore, represent the best evaluation that can be obtained by ESD-STM of the masses of these polymers. The measures of uncertainty for the STM, KM, and parametric approaches given in Table 1 were calculated using bootstrapping. The uncertainty given is the sample standard deviation of the corresponding quantities calculated from 200 nonparametric bootstrap data sets.

The STM-derived *M*_n_, *M*_w_, and DP_n_ values can be directly compared to the results obtained from NMR (Table 1). For nominally identical polymers P1 and P2, the NMR values agree very well with the STM ones. This excellent comparison allows us to benchmark the ESD-STM approach also for the characterization of mean mass values, while showing that this technique is not affected by the same limitations of NMR. This is directly demonstrated by the results obtained by ESD-STM for P3, that have the same accuracy as those for P1 and P2, but for which high quality NMR spectra were not available (Figure S9). Finally, we notice that having access to the full mass distributions allows one to use the STM data to further characterize these polymers in ways that are inaccessible by NMR. In fact, from the fitting procedure by means of a Flory-Schultz distribution and a logistic curve, different parameters can be derived (such as an effective fractional monomer conversion, see Section 7 of the SI) that provide an accurate and specific description of the mass distribution.

Full mass distributions can theoretically be obtained also by SEC, although in the specific case of conjugated polymers the aforementioned intrinsic limitations and inaccuracies of SEC caused by polystyrene calibration and aggregation severely limit its reliable use. Nevertheless, SEC is still often used in the literature as an “effective characterization tool”, and we thus decided to compare its outcomes with the ESD-STM results, for completeness. SEC was employed for all three samples to obtain the *M*_n_ values that are reported in Table 1 (see Section 10 of the SI for details). The *M*_n_ values determined by SEC for P1 and P2 are in the same relationship as the values determined by ESD-STM, i.e., *M*_n,P1_ > *M*_n,P2_, but this is not true for P3, for which SEC gives significantly larger *M*_n_ values than for P1 and P2, while the ESD-STM data show that *M*_n,P3_ is comparable to the *M*_n_ of P1 and P2. Moreover, for all three polymers, the absolute *M*_n_ values obtained by SEC are significantly larger than the corresponding ESD-STM ones.

**Figure 4:**
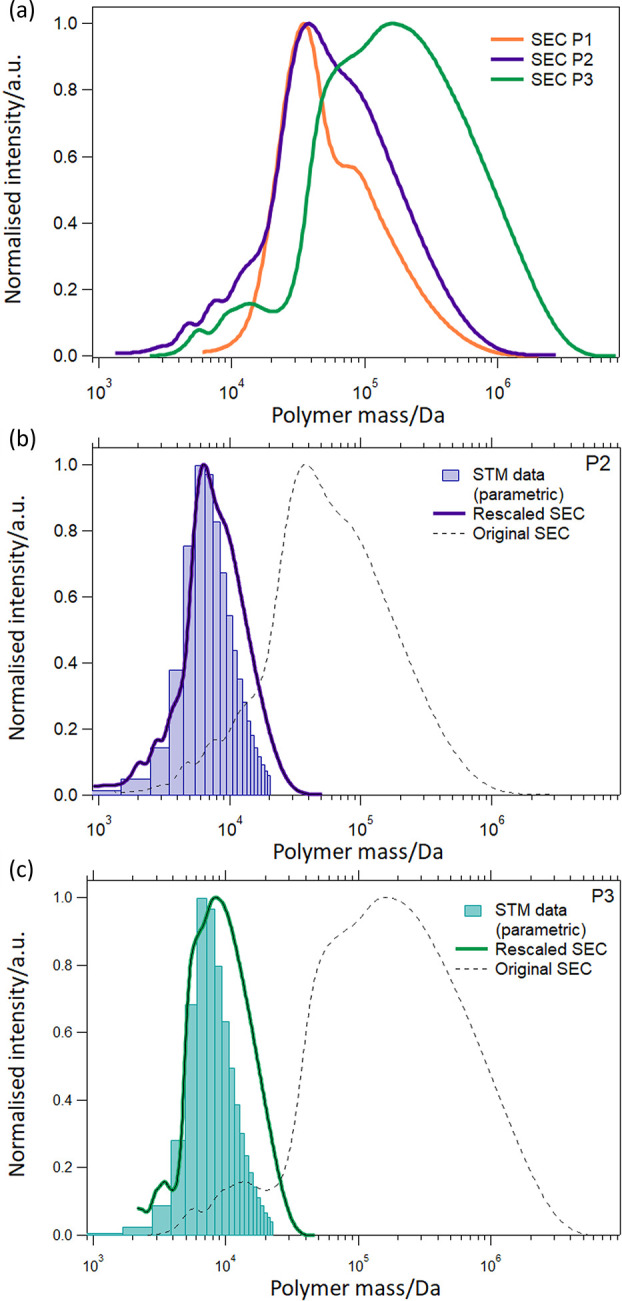
(a) SEC mass distributions for the three polymers. P1 and P2 have similar average masses and aggregation shoulders in similar positions; on the contrary, P3 displays a much wider aggregation shoulder caused by its longer and more flexible side chains promoting aggregation. After the recalibration of the SEC horizontal mass scales, the comparison with the STM data (corrected through the parametric survival approach) demonstrates an excellent agreement, as shown in (b) and (c) for P2 and P3, respectively. The STM results are shown as histograms, the rescaled SEC data as continuous lines and the original SEC data as dotted black lines.

This becomes even more evident when directly comparing the full mass distributions derived from the STM analysis with those obtained by SEC: the well-known issue of the overestimation of the SEC masses^20,21,23,27^ is clearly observable, as can be seen in Figure S13b–d for P1, P2, and P3, respectively. However, a careful look at the SEC curves (Figure 4a) reveals that oligomer peaks, which were probably not completely removed during Soxhlet extraction, are visible in the low-mass regions of P2 and P3. Using these peaks as a reference, it is possible to evaluate the functional relationship between the SEC measured masses (determined though universal calibration using the viscometer detector) and their actual values (see Figure S14). The inverse of this functional relationship can then be used to rescale the full SEC mass distribution. Figure 4b,c shows the results of applying this procedure to the original SEC data for P2 and P3, respectively, and the direct comparison with the STM data now demonstrates a much better agreement between the two techniques. Note that the STM data are reported as histograms and not as continuous distributions since they are based on counts of integer multiples of the polymer repeat unit and thus their *x*-axis scale is discrete. The aggregation shoulder is clearly still present also in the rescaled SEC data, as it represents an intrinsic feature of the SEC measurement. However, this shoulder is not present in the STM histograms, since even longer polymer strands can be clearly identified and do not suffer issues of aggregation after the ESD process. The fact that, for both polymers, a quadratic functional dependence relates the SEC masses and the theoretical values (see Figure S14), could represent a general trend in the calibration of SEC data of conjugated polymers, and will be investigated in more detail in the future. At the same time, these results indicate that the ESD-STM technique is potentially an ideal way to recalibrate SEC data for conjugated polymers.

Another essential characteristic of conjugated polymers that can influence the properties of devices is the type and frequency of chemical defects that alter the ideal sequence and that are caused by side reactions during polycondensation.^35,37,43,44^ In the case of PThDPPThF4, CH/CH hc reactions of the ThDPPTh monomer have already been verified by ^1^H NMR spectroscopy.^34^ Specifically for the polymers investigated here, end group and defect integration of ^1^H NMR peaks assigned to ThDPPTh hc defects provides a relative frequency of (6.2 ± 0.5)% and (2 ± 1)% for P1 and P2, respectively (see Figures S7 and S8 and Table 2). This proves that the different synthetic conditions used for these two polymers (see theSI) indeed produce different hc defect content. Similar to what already discussed for the NMR evaluation of the *M*_n_ values, it is not possible to identify or quantify hc defects for P3, since its much stronger aggregation tendency causes a significant broadening of the peaks in the ^1^H NMR spectra (see Table 2 and Figure S9).

**Table 2:** Homocoupling Defect Content for P1, P2, and P3 as Measured by STM and NMR

polymer	STM (%) (total # counts)	NMR (%)
P1 (*x* = 0)	5.9 ± 1.0 (547)	6.0 ± 1.0
P2 (*x* = 0)	3.0 ± 0.8 (434)	2 ± 1
P3 (*x* = 4)	6.7 ± 1.0 (551)	--

**Figure 5:**
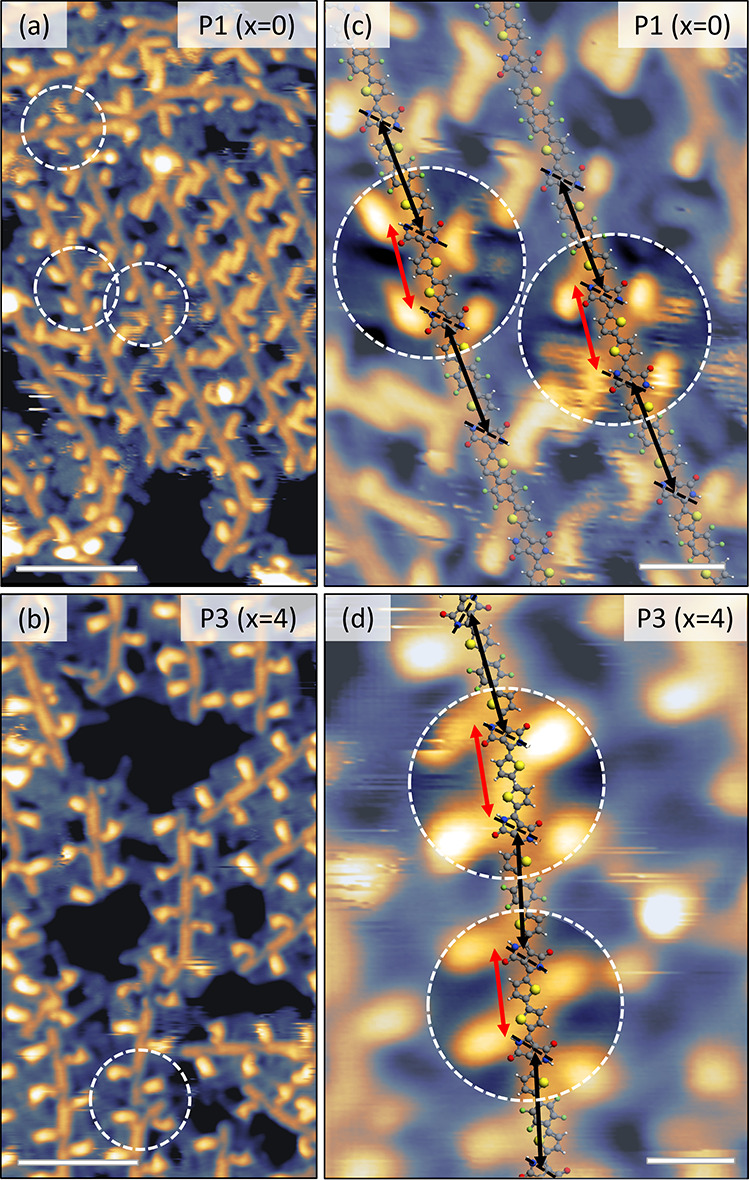
Sequencing the backbones of the polymers allows for the precise identification of homocoupling defects. (a) and (b) show the same STM images of Figure 1, with white dashed circles indicating the positions of ThDPPTh homocoupling defects. (c, d) Higher-resolution STM images with examples of homocoupling defects highlighted by white circles. The red and black double arrows indicate the distance along the backbones between two successive side chain exit points, showing that in the case of a homocoupling (red double arrows), this quantity is significantly reduced with respect to the regular heterocouplings (black double arrows). Scale bars correspond to 4 nm for parts (a) and (b) and to 1 nm for parts (c) and (d). The STM images were acquired in constant current mode, with tunneling parameters (a) 1.4 V, 70 pA; (b) 1.3 V, 90 pA; (c) 1.4 V, 70 pA; (d) 1.3 V, 90 pA.

The presence of ThDPPTh hc defects can also be determined from the ESD-STM data, as the side chains are well visible and their separation along the backbone is significantly shorter in the case of an hc defect than for a normal heterocoupling (see Figure 5 and Figure S5). A careful analysis of high-resolution STM images indeed confirms the presence of ThDPPTh hc in all studied polymers, as shown in Figures 5c,d for P1 and P3, respectively, and in Figure S5b for P2. To verify this quantitatively, the polymer backbones were fitted for a large number of STM images (see Section 3 of the SI for details), resulting in an average value of (1.21 ± 0.09) nm for the shorter distances between successive side chains and (1.62 ± 0.07) nm for the longer ones. The comparison between these values and the corresponding theoretical ones obtained from optimized molecular models is highly satisfactory (Table S1), unambiguously proving the nature of the hc defects. By analyzing a large number of STM images, the hc content was quantified through this approach for all three polymers and reported in Table 2. In the case of P1 and P2, for which the corresponding NMR-based values are available, the agreement is excellent, representing a reliable benchmarking of the ESD-STM technique. Since for P3 the ^1^H NMR signals are broad and ill-resolved, its hc content can only be determined based on STM imaging, demonstrating how this method reaches beyond the limits of current analytical techniques. We also observe that the overall trend of hc defects obtained from the STM analysis is confirmed by UV–vis measurements, where the intensity of the hc shoulder at about 750 nm, is similar for P1 and P3, but lower for P2 (Figure S15).^34^ It should be further noticed that the uncertainties associated with the STM measurements are purely statistical in nature, as they are estimated as the standard deviation of a binomial distribution with *n* trials (*i*.*e*., total number of bonds counted) and success probability *p* (that is the probability of having a correct, nondefective bond between successive monomers). These errors could, therefore, be further reduced by increasing the number of analyzed polymers. On the contrary, errors associated with the NMR measurements are inherently linked to the resolution of the spectra, and therefore cannot be further minimized. Finally, it is important to note that while the signal obtained from spectroscopic techniques only depends on the average frequencies of the polymerization defects, ESD-STM grants access to the entire distribution of backbone sequences. This allows one to investigate specific characteristics of the defects such as preferential locations within the backbones (e.g., in correspondence to end groups) or possible spatial correlations between defects. This kind of information is mostly inaccessible to traditional analytical techniques but represents a fundamental knowledge in order to determine how defects affect the catalytic cycle, e.g., if they increase the probability of side reactions or enhance the chances of terminating the polymerization process. Future research will explore this specific insight offered by the ESD-STM technique.

## Conclusions

In this work, we demonstrate that ESD-STM is a reliable analytical methodology for characterizing conjugated polymers by showing that it can accurately and quantitatively reproduce the results of traditional analytical techniques where they are applicable, while providing equivalent precision in cases where these methods are inadequate. Furthermore, we establish its ability to deliver microstructural information that cannot be obtained by any other current analytical technique, such as molecularly resolved assembly patterns, backbone conformations, exact sequencing, nature and frequency of polymerization defects, and full mass distributions of the macromolecules. The ESD-STM benchmarking was performed by measuring three low-molecular-weight DPP conjugated polymers synthesized by DArP (two with the same structure but different homocoupling content, the third one with the side chain branching point farther away from the backbone) and comparing the results with the outcomes of GIWAXS, solid-state NMR, NMR, and SEC.

The average separation between backbones determined by analyzing a large number of STM images showed excellent agreement with the lamellar separation derived from GIWAXS measurement. Furthermore, otherwise inaccessible details of the molecular assembly were obtained by fitting geometry-optimized molecular models onto high-resolution STM images. These revealed the submolecular characteristics of the interdigitation patterns between polymer side chains, which are driven by the maximization of van der Waal interactions. Moreover, the same analysis allowed us to establish that the conformation of the backbones is characterized by N,S-*anti* configurations between DPP and neighboring thiophene units, in excellent agreement with the results of solid-state NMR spectroscopy and DFT calculations.^57,61^

By measuring the length of a large number of individual polymer molecules in wide-scan STM images and renormalizing the ensuing distribution via a survival analysis approach, we determined the polymer length (mass) distributions. The corresponding *M*_n_ values agree extremely well with those obtained by integrating the NMR spectra for the two polymers where the baseline resolution is high enough for this to be possible. However, ESD-STM can also determine, with the same degree of accuracy, the mass distribution for the third polymer whose higher aggregation tendency causes broadening of the NMR spectra and prevents a reliable integration of the end-group signals. A further advantage of ESD-STM is that it establishes the full mass distribution as opposed to only average mass values as obtained from NMR. This gives access to much richer data about the polymers and allows us to derive direct information on the synthetic pathway by which they were made. The decision to focus this study on low *M*_n_ conjugated polymers stemmed from the objective of benchmarking the ESD-STM technique against traditional analytical methods like NMR. This necessitated working with low *M*_n_ polymers, as NMR can only achieve adequate baseline resolution if chains can be solubilized sufficiently well. This requires either high solubility or a short chain length. On the other hand, many high-performance polymers of practical relevance show strong aggregation and thus broad signals in ^1^H NMR spectra even for short lengths. It is therefore crucial to recognize that a limited molecular weight analysis pertains to NMR and not ESD-STM. Indeed, our previous studies have demonstrated ESD-STM’s capability to measure and analyze much longer conjugated polymers (for example IDT-BT-C16 polymers with *M*_n_ exceeding 50 kDa).^52^

An internal calibration procedure was proposed for rescaling the SEC mass distributions of the analyzed polymers, based on the signals of short oligomers. The agreement of the resulting, corrected distributions with those determined by ESD-STM was excellent in the low and medium mass range, further proving the reliability of ESD-STM. In the higher mass range, SEC distributions showed the well-known aggregation shoulders, which could be directly evaluated by comparison with the ESD-STM distribution, since this latter technique is not affected by polymer aggregation.

Finally, the modeling of high-resolution STM images also allowed us to determine the nature and to quantify the occurrence of homocoupling defects. When compared with the NMR analysis of the two polymers with shorter side chains, the agreement was again excellent, both in terms of the type of homocoupling (only ThDPPTh hc were observed by both techniques) and of their average frequencies. Similar to the case of the average molecular mass, while the NMR analysis cannot be performed on the third, more aggregating polymer, ESD-STM is not affected by polymer aggregation and thus also provides results on the homocoupling defects of the polymer with the longer side chains. It is to be noted that, while the degree of uncertainty associated with the NMR estimation of the average mass values and the defect frequencies depends on the specific polymer and on the quality of the spectrum, ESD-STM affords the same accuracy in different measurements, provided that a similarly sized statistical sample is analyzed.

Overall, this work showcases the power of ESD-STM as an analytical tool for the characterization of conjugated polymers, capable of providing a wealth of high-resolution quantitative information that is inaccessible to traditional analytical techniques. Particularly attractive are its sensitivity to the presence of minimal quantities of chemical defects or specific molecular conformations and the fact that, being a microscopy technique, it allows access to information about individual members of a sample population. Thus, at variance with more traditional, population-integrating spectroscopy techniques, the information that can be extracted from ESD-STM also includes correlations within populations (e.g., spatial correlations of polymerization defects), which give a previously unattainable insight into the synthetic pathways through which these macromolecules are made and a way to test hypotheses on reaction mechanisms. Thus, we expect that ESD-STM will deliver a fundamental contribution toward the rational optimization of the synthesis of conjugated polymer materials with tailored and improved functional properties.

## Methods

### Nuclear Magnetic Resonance (NMR)

Polymers were measured on a Bruker AVANCE III 500 spectrometer (1H: 500.1 MHz, 13C: 125.8 MHz). C_2_D_2_Cl_4_ (at 120 °C) was used as a solvent. The spectra were referenced to the residual solvent peak (C_2_D_2_Cl_4_: δ(1H) = 5.98 ppm). Monomers and other compounds were measured on a Bruker Avance 300 instrument (1H NMR at 300 MHz and 13C NMR spectra at 75 MHz at room temperature in CDCl3 (δ(1H) 7.26 ppm; δ(13C) 77.0 ppm).

### UV–vis Spectroscopy

UV–vis spectra were recorded at 25 °C on a UV-1800 Series (Shimadzu) instrument equipped with a heated sample holder in 2-chloronaphthalene solutions (*c* = 0.02 mg/mL) at 150 °C.

### Size Exclusion Chromatography (SEC)

The polymers were measured with an Agilent Infinity II MDS instrument with differential refractive index (DRI), viscometry (VS), dual angle light scatter (LS), and multiple wavelength UV detectors. The system was equipped with 2 x PLgel Mixed C columns (300 mm × 7.5 mm) and a PLgel 5 μm guard column, with CHCl_3_ as the eluent. Samples were run at 1 mL/min at 30 °C. Poly(methyl methacrylate) and polystyrene standards (Agilent EasiVials) were used for calibration. Ethanol was added as a flow rate marker. Analyte samples were filtered through a nylon membrane with a 0.22 μm pore size before injection. Experimental molar mass (*M*_n_) and dispersity (*Đ*) values of the synthesized polymers were determined by conventional calibration using Agilent GPC/SEC software.

### Scanning Tunnelling Microscopy (STM)

STM measurements have been performed on a low temperature (LT) ultrahigh vacuum (UHV) scanning tunnelling microscope equipped with an electrospray deposition (ESD) setup. The three polymer samples (P1, P2, and P3, see main text for definition) were dissolved in chlorobenzene at concentrations ∼ 0.025 g/L, and methanol was added to a 4:1 volume ratio. The molecules were deposited from solution by ESD with a 4-stage Molecular Spray system on a room temperature Au(111)/mica sample. The deposition current was monitored on the target substrate and typical total deposition charges were 4 pAh. Gold on mica thin films (Georg Albert PVD, 300 nm thickness) were used as substrates and prepared in UHV by cycles of argon ion sputtering (1 kV) and subsequent annealing to 500 °C. The LT-STM (CreaTec Fischer & Co. GmbH) was kept at −196 °C by a liquid nitrogen bath cryostat during measurements. All images were acquired in constant current feedback mode with an electrochemically etched tungsten tip that was treated by argon ion sputtering after insertion in UHV. The bias voltage indicated in the figure captions was applied to the sample. The STM images were processed with WSxM^65^ and Gwyddion,^66^ the molecular models were made and optimized in Avogadro^67^ and their fitting to the STM images was performed with the LMAPper software.^68^

## Data Availability

The datasets generated during and/or analyzed during the current study are available from the corresponding author on reasonable request.

## Supporting Information Available

Further details on the experimental and synthetic methods, STM images displaying different molecular coverages and the assembly and modeling of P2 polymers, molecular models and procedure used for fitting the STM images, details of the quantitative analysis of the NMR spectra, procedure for determining polymer length profiles from STM images, survival analysis method used to correct the polymer length distributions, discussion of effective extents of reaction, SEC analysis details, UV–vis spectroscopy data, method for calculating average values from distributions (PDF)
