# Supplementary material for: Molecular-Scale Imaging Enables Direct Visualization of Molecular Defects and Chain Structure of Conjugated Polymers
Source: ACS Nano. 2024 Apr 23;18(18):11655–64. doi: 10.1021/acsnano.3c10842 (PMC11080458; doi:10.1021/acsnano.3c10842)
Supplement: Supplementary file 1 — nn3c10842_si_001.pdf [file nn3c10842_si_001.pdf]

Supplementary Information

Molecular-Scale Imaging Enables Direct Visualization of Molecular Defects and Chain Structure of Conjugated Polymers

Stefania Moro[1,2], Simon E.F. Spencer[3], Daniel W. Lester[4], Fritz Nübling[5], Michael Sommer[6,7], Giovanni Costantini[1,2]*

[1]*School of Chemistry, University of Birmingham, Birmingham B15 2TT, UK*

[2]*Department of Chemistry, University of Warwick, Coventry, CV4 7AL, UK*

[3]*Department of Statistics, University of Warwick, Coventry, CV4 7AL, UK*

[4]*Polymer Characterisation Research Technology Platform, University of Warwick, Coventry, CV4 7AL, UK*

[5]*Institute for Macromolecular Chemistry, University of Freiburg, 79104 Freiburg, Germany*

[6]*Institute for Chemistry, Chemnitz University of Technology, 09111 Chemnitz, Germany*

[7]*Center for Materials, Architectures and Integration of Nanomembranes (MAIN), Chemnitz University of Technology, 09126 Chemnitz, Germany*

Table of Contents

Experimental section 2

Synthesis and materials 2

Monomer synthesis 2

Polymer synthesis 5

1. Different molecular coverages 6
2. Assembly and modelling of P2 polymers 7
3. Modelling and STM images fitting procedure 8
4. NMR analysis details 10
5. Determining polymer length profiles from STM images 13
6. Corrections to length distributions 15
7. Effective extents of reaction 21
8. SEC analysis details 22
9. UV-vis spectroscopy 25
10. Calculating average values from distributions 26

References 27

Experimental section

Synthesis and materials

F_4Br_2, $Pd(OAc)_2$, Pd_2dba_3, solvents, ligands, bases and pivalic acid were obtained from Sigma Aldrich and used as received. Solvents were individually degassed prior to use in DArP. All other chemicals were obtained from Sigma Aldrich and used as received unless otherwise noted.

Monomer synthesis

Iodide **2**, ThDPPTh **5**, and the DPP monomers with 2-octyldodecyl side chains for P1 and P2 were made according to the literature.[1] The ThDPPTh monomer **6** with x = 4 for P3 was made starting from 2-octyldodecanol as commercial starting reagent as shown in **Scheme S1**.

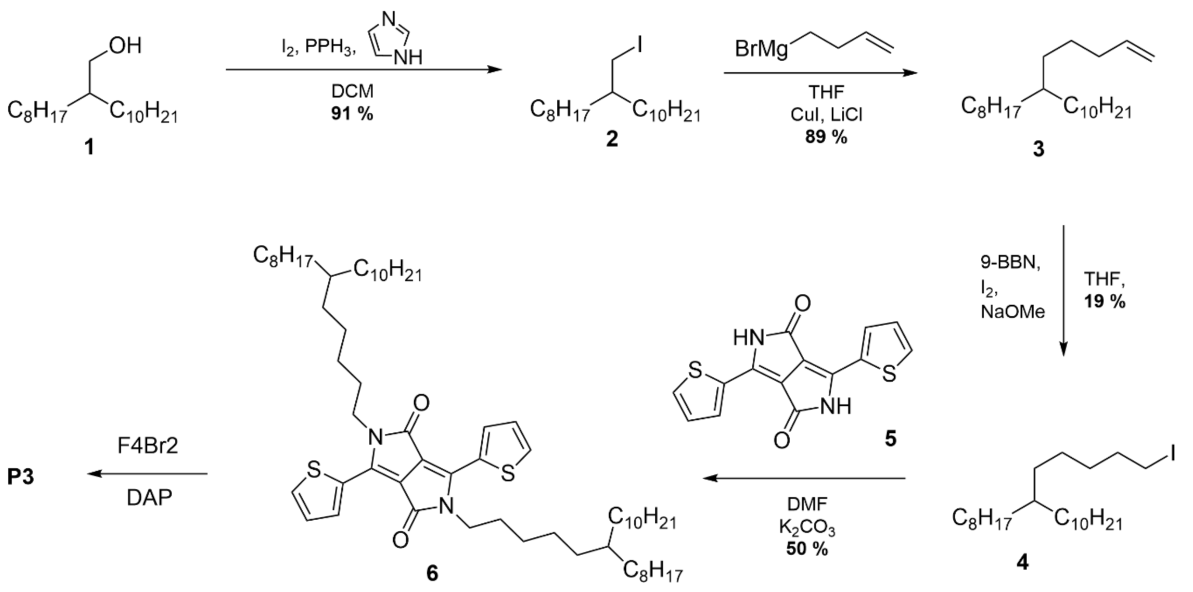

Scheme S1. Overview of synthesis of monomer 6 with extended linker (x = 4).

Synthesis of 9-(pent-4-enyl)nonadecane ***3***

To a flame-dried 250 mL Schlenk flask under N_2 atmosphere CuI (0.57 g, 3.0 mmol), LiCl (1.27 g, 30.0 mmol), 40 mL dry THF and 9-(2-iodomethyl)nonadecane **2** (6.12 g, 15.0 mmol) were added and cooled to 0 °C. To the mixture, a solution of 3-butenylmagnesium bromide (1.6 M in THF, 11.3 ml, 18.0 mmol) was dropwise added during 15 min, and the whole stirred for 18 h at 0 °C. The mixture was allowed to warm to RT, and a saturated aqueous solution of NH_4Cl (120 ml) was added. The organic phase was separated, the aqueous phase extracted three times with diethyl ether (3 × 120 ml) and the organic phases combined and washed with saturated brine (2 × 150 ml) and dried over $MgSO_4$. The solvents were removed under reduced pressure. The remaining liquid was diluted with *n*-hexanes and filtered over a short plug of silica gel. The solvent was removed under reduced pressure and **3** was obtained as a colourless oil. Yield: 4.5 g, 13.4 mmol, 89 %.

^{1}H-NMR (300 MHz, $CDCl_3$): δ = 5.80 (ddt, 1H, ^3J = 6.00 Hz, ^3J = 9.00 Hz, ^3J = 18.00 Hz, 4‘-H), 5.04-4.87 (m, 2H, 5‘-H_2), 2.10-1.95 (m, 2H, 3‘-H_2), 1.40-1.17 (m, 35H, 1‘-2‘-H_2, 9-H, 2-8-H_2, 10-18-H_2), 0.96-0.80 (m, 6H, 1-H_3, 19-H_3) ppm.

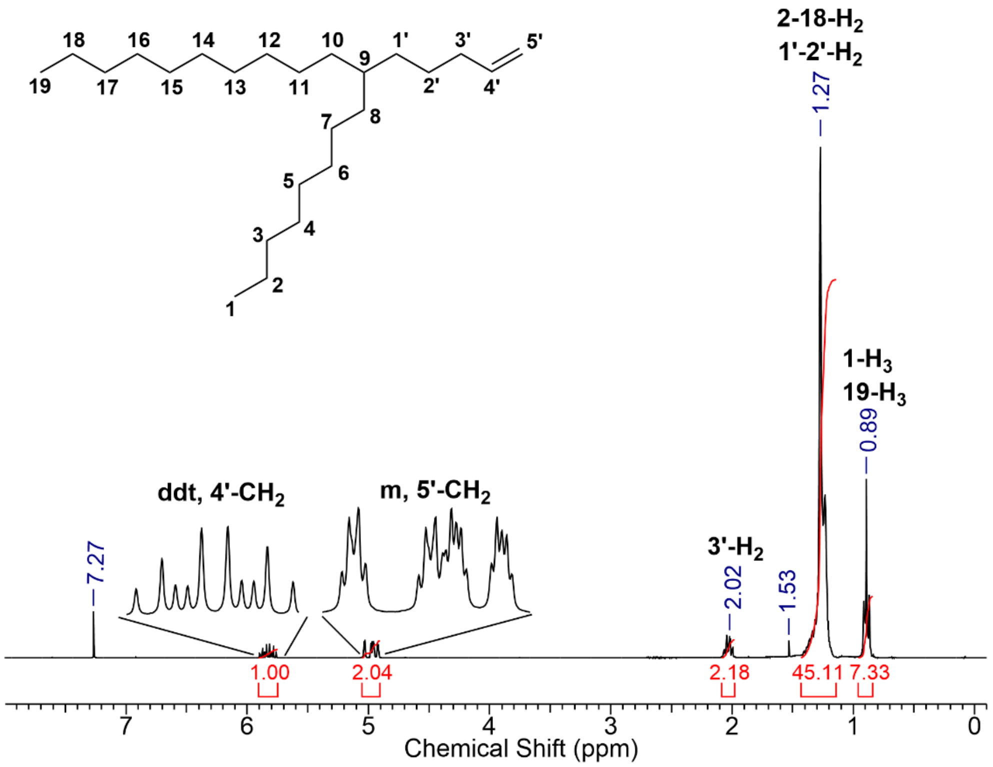

Figure S1. ^{1}H NMR spectrum of **3** in $CDCl_3$.

Synthesis of 9-(5-Iodopentyl)nonadecane ***4***

To a flame-dried 250 mL Schlenk flask under N_2 atmosphere **3** (2.60 g, 7.73 mmol) and a solution of 9-BBN in THF (0.5 M, 17 mL, 8.5 mmol) were added and stirred at RT for 16 h. Methanol (0.05 mL) was added, the mixture was cooled to 0 °C, and I_2 (2.75 g, 10.82 mmol) was added in portions. A solution of NaOMe in methanol (5.4 M, 2.01 mL, 10.82 mmol) was added dropwise within 15 min, and the whole was stirred for 17 h at RT. A saturated solution of $Na_2S_2O_3$ (10 mL), H_2O (20 mL) and *tert*-butyl methyl ether (50 mL) were added subsequently. The organic phase was separated, the aqueous phase extracted with *tert*-butyl methyl ether (2 × 50 mL), the organic phases combined and dried over $MgSO_4$. The remaining solution was filtered over a short plug of silica gel and the solvents were removed under reduced pressure. **4** was obtained as a colourless oil. Yield: 0.545 g, 1.17 mmol, 15 %.

^{1}H-NMR (300 MHz, $CDCl_3$): δ = 3.20 (t, 2H, ^3J = 7.13 Hz, 5‘-H_2), 1.81 (quin,2H, ^3J = 6.96 Hz, 4‘-H_2), 1.40-1.17 (m, 37H,9-H, 1‘-3‘-H_2 2-8-H_2, 10-18-H_2), 0.96-0.80 (m, 6H, 1-H_3, 19-H_3) ppm.

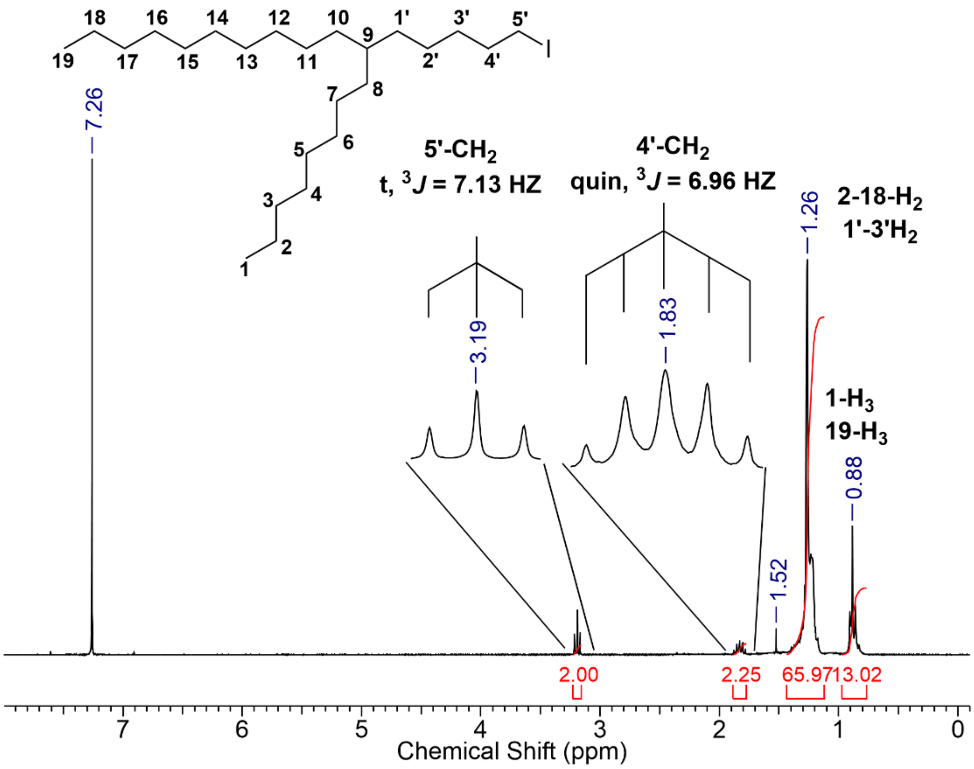

Figure S2. ^{1}H NMR spectrum of **4** in $CDCl_3$.

Synthesis of 2,5-di((6-octyl)hexadecane)-3,6-dithiophen-2-ylpyrrolo[3,4-c]pyrrole-1,4-dione **6**

To a flame-dried 125 mL Schlenk flask under N_2 atmosphere dried K_2CO_3 (270.9 mg, 1.96 mmol) and ThDPPTh **5** (128.70 mg, 0.49 mmol) were suspended in dry DMF (10.0 mL) and stirred for 2 h at 120 °C. Within 4 h, **4** (0.7 g, 1.5 mmol) was added dropwise as a solution in dry DMF (1 mL), and the mixture was stirred for 20 h at 120 °C. The mixture was cooled, the solvent removed and the residue taken up in DCM and filtered over a plug of silica gel. The raw product was purified on a silica gel column (SiO_2, $CHCl_3$:*i*-Hex / 1:1 / v:v, R_f = 0.55) and obtained as dark-red solid. Yield: 272 mg (0.28 mmol, 57 %).

^{1}H-NMR (300 MHz, $CDCl_3$): δ = 8.95-8.91 (dd, 2H, ^3J= 3.92 Hz, ^4J = 1.20 Hz, 3‘‘-H_2), 7.65-7.61 (dd, 2H ^3J = 5.05 Hz, ^4J = 1.20 Hz, 1‘‘-H_2), 7.31-7.27 (dd, 2H ^3J = 5.05 Hz, ^4J = 3.92 Hz, 2‘‘-H_2), 4.11-4.03 (m, 4H, 5’H_2), 1.80-1.65 (m, 4H, 4‘-H_2), 1.40-1.15 (m, 74H, 2-18-H_2, 1‘-3‘-H_2), 0.95-0.77 (m, 6H, 1-H_3, 19-H_3) ppm.

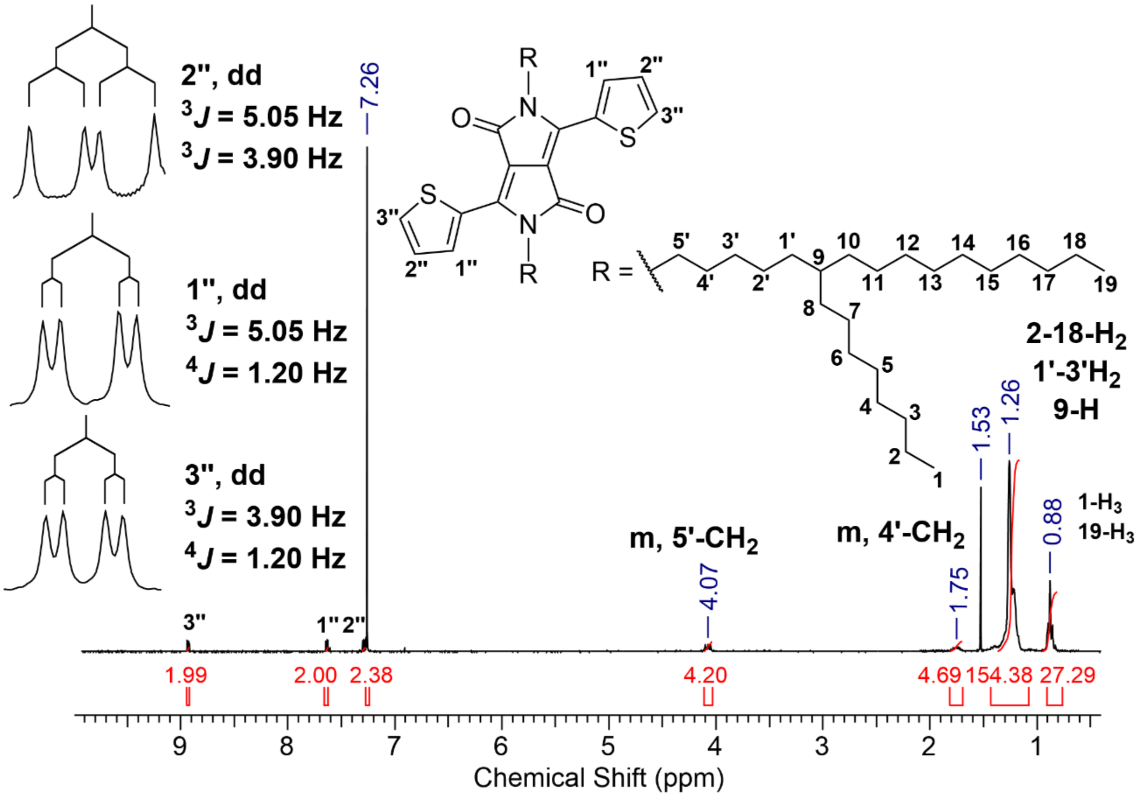

Figure S3. ^{1}H NMR spectrum of **6** in $CDCl_3$.

Polymer synthesis

P1 was prepared by adding 86.0 mg ThDPPTh with 2-octyldodecyl side chain (0.1 mmol), 31.0 mg F_4Br_2 (0.1 mmol) and 10.2 mg PivOH as stock solutions in toluene into a screw-cap vial containing a stir bar. The toluene was evaporated to dryness. 41.5 mg K_2CO_3 (0.3 mmol), 0.167 mL degassed DMAc and 0.167 mL degassed toluene were added. 0.9 mg $Pd(OAc)_2$ (0.004 mmol) were added carefully and the mixture was purged with nitrogen for a short time. The vial was sealed and stirred at 90 °C for 72 h. The mixture was cooled, diluted with chloroform, precipitated into methanol, filtered and subjected to Soxhlet extraction with methanol, ethyl acetate, and chloroform. The chloroform fraction was obtained as blue-green solid. Yield 65%, $M_{n,SEC}$ = 41.5 kg/mol, Đ = 2.13.

P2 was prepared by adding 86.0 mg ThDPPTh with 2-octyldodecyl side chain (0.1 mmol), 31.0 mg F_4Br_2 (0.1 mmol), 10.2 mg PivOH and 2.43 mg P(*o*-anisyl)$_3$ (0.008 mmol) as stock solutions in toluene into a screw-cap vial containing a stir bar. The toluene was evaporated to dryness. 98 mg Cs_2CO_3 (0.3 mmol) and 0.2 mL degassed toluene were added. 1.83 mg Pd_2dba_3 (0.002 mmol) were added carefully and the mixture was purged with nitrogen for a short time. The vial was sealed and stirred at 100 °C for 72 h. The mixture was cooled, diluted with chloroform, precipitated into methanol, filtered and subjected to Soxhlet extraction with methanol, ethyl acetate, and chloroform. The chloroform fraction was obtained as blue-green solid. Yield 85%, $M_{n,SEC}$ = 31.6 kg/mol, Đ = 3.27.

P3 was prepared in the same way as **P2** but with **6** as comonomer. Yield 88%, $M_{n,SEC}$ = 74 kg/mol, Đ = 4.85.

1. Different molecular coverages

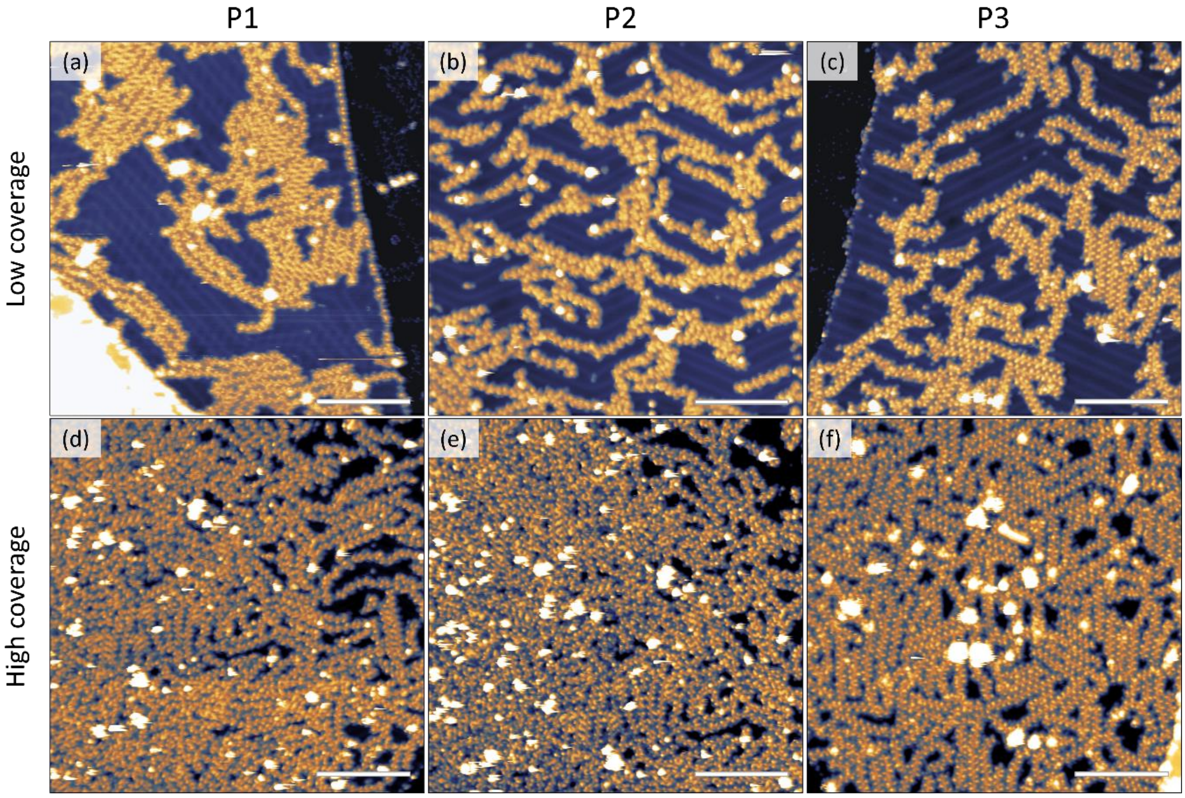

Figure S4. STM images showing the tendency of the P1, P2 and P3 polymer to interact with each other both at low molecular coverage in (a), (b) and (c), respectively, and at high molecular coverage in (d), (e) and (f), respectively. Scale bars correspond to 20 nm. The STM images were acquired in constant current mode with tunnelling parameters (a) 1.7 V, 70 pA; (b) 1.2 V, 120 pA; (c) 1.1 V, 100 pA; (d) 1.3 V, 70 pA; (e) 0.75 V, 90 pA; (f) 1.1 V, 100 pA.

2. Assembly and modelling of P2 polymers

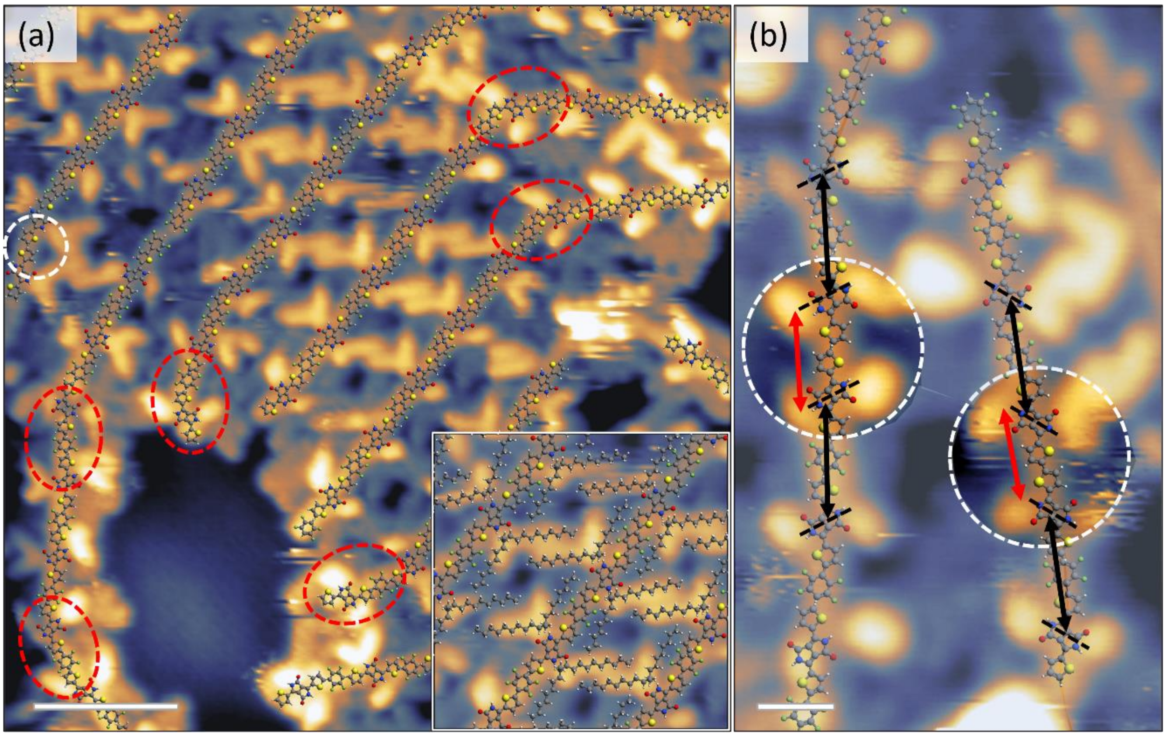

Figure S5. STM images for the polymer P2, equivalent to **Fig. 1**, **2** and **5** for P1 and P3. (a) High-resolution STM image showing the assembly patterns of P2. Molecular models for the backbones are overlayed onto the STM image. White circles identify homocoupling defects, red ellipses identify N,S-*anti* configurations of the DPP group with respect to the neighbouring thiophene unit, resulting in a bend of the backbone. In the inset, the side chain assembly pattern is shown. (b) High-resolution examples of homocoupling defects for the polymer P2, resulting in shorter distances (red double arrows) when compared to those corresponding to standard heterocouplings (black double arrows). Scale bars correspond to 3 nm in (a) and 1 nm in (b), respectively. The lateral size of the inset in (a) is 5 nm. The STM images were acquired in constant current mode with tunnelling parameters (a) 1.4 V, 70 pA; (b) 0.9 V, 120 pA.

3. Modelling and STM images fitting procedure

Geometry optimised molecular models were created in the Avogadro molecular editor for both the backbones and the side chains[2]. In order to model the backbones, the two co-monomers (*i.e.*, the tetrafluorobenzene and the ThDPPTh units) have been separately built in Avogadro and energy minimised by using the MMF94 force field. For the ThDPPTh units, different N,S-*syn* or -*anti* configurations of the thiophene rings with respect to the neighbouring DPP group have been created, in order to account for the possibility of rotations around the connecting C-C single bonds.

The geometry optimised models were then rescaled to the size of the STM images in LMAPper.[3] Exploiting the positions where the sidechains are connected to the backbones (characterised by bright dots in the STM images as explained in the main text), the DPP units were univocally identified in the images. The choice of the -*syn*/-*anti* conformation of the thiophenes within the ThDPPTh unit was dictated by the best fit to the local backbone conformation. Tetrafluorobenzene units were then added in the spaces between the thiophenes of different subunits. If there was no space left, a homocoupling defect was counted. To further verify that these features actually corresponded to a polymerisation defect in a quantitative way, in the case of P1, the distance between the positions where the sidechains connect to the backbone was measured along the backbone. The values measured for all homocouplings identified in the fitting procedure gave an average distance of (1.21 ± 0.09) nm, while the average distance obtained for an equal number of heterocouplings was (1.62 ± 0.07) nm. These values both match very well the values obtained from the Avogadro models, as shown in **Table S1**.

Table S1. Distance between successive positions where the side chains are connected to the backbone in the case of heterocouplings and homocouplings for P1, showing a significantly shorter distance in the case of defects. Reference values obtained from models made in Avogadro are also reported, showing a very good agreement.

Sequence	Model	Experimental (STM) / nm	Model (Avogadro) / nm
**regular (hetherocoupling)**	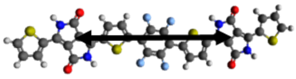	1.62 ± 0.07	1.67
**defective (ThDPPTh homocoupling)**	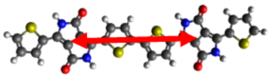	1.21 ± 0.09	1.25

An example of the backbone fitting is shown in **Fig. S6**, where white dashed circles indicate homocouplings of the ThDPPTh units and red dashed ovals indicate N,S-*anti* configurations in the sequence of the polymers. Even though the N,S-*syn* configuration of the thiophene with respect to the neighbouring DPP unit is the most common one,[4] N,S-*anti* conformations were also seen to occur, both at the end of the polymers and within them, causing significant disruptions to the local assembly. The loss of intermolecular interaction induced by these conformational defects has been reported to affect the overall backbone conformation also in other polymer systems.[5]

Higher resolution images were typically needed in order to understand the side chain interdigitation patterns, as these require details of the side chains themselves to be clearly distinguishable. As discussed in the main text, the linker section of the sidechains (**Fig. 1a** in the main paper), is usually seen in the STM images as a bright feature on both sides of the backbones. To model the two arms, instead, several

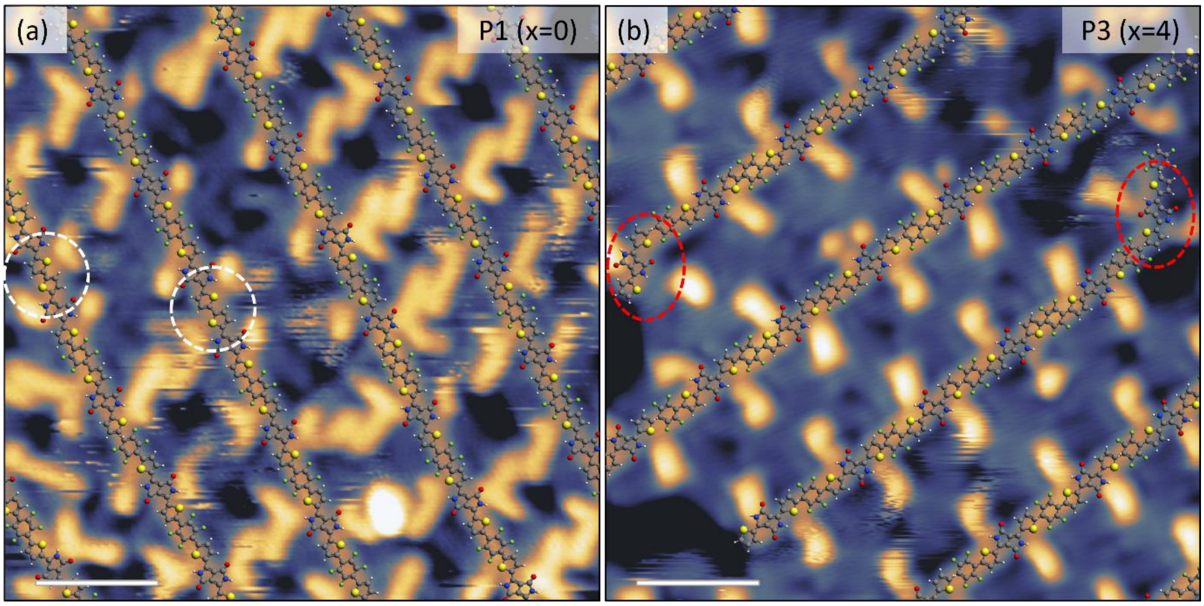

Figure S6. (a) and (b) show the result of the backbone fitting process on a larger scale. White dashed circles identify homocoupling defects along the backbones, while red dashed ellipses represent the N,S-*anti* configurations of the DPP group with respect to the neighbouring thiophene unit. Such conformational defects cause a bend of the backbone. Scale bars correspond to 2 nm. The STM images were acquired in constant current mode with tunnelling parameters (a) 1.4 V, 70 pA; (b) 1.3 V, 140 pA.

different molecular models were first created to identify all possible conformations. Fully optimised sidechains were then built in Avogadro and fitted to the images. This process has been iteratively repeated until the fit was satisfactory and it was possible to account for all features visible in the STM images.

These more complex molecular models represent the STM data very well in the ordered parts of the images. In more disordered areas of the sample (as is often the case at the borders of molecular island) or when backbone sequence defects are present, the side chains adopt random configurations that are seemingly based on the local available space. As mentioned in the main text, in ordered areas the side chains of P1 and P2, are seen to form regular patterns, with apparent heights that smoothly vary from high (very bright in the STM images, closer to the linker) to low (darker, towards the end of the arms). In disordered areas of the sample, instead, all parts of the side chains are seen to be generally dimmer in intensity, aside from the bright linker. The hypothesis we propose to account for this behaviour is that in regular areas the density of the side chains is such to promote a more 3D assembly. In fact, due to the presence of sp^3 carbons in the side chains, for polymers adsorbed on a surface, Carbon atoms close to the connecting position of the side chains to the backbone will be sterically forced away from the substrate (a similar behaviour has already been seen for a different DPP-based polymer, see Ref.[6]). The result is that the initial part of the side chains, the one closer to the backbone, will be higher than the backbones which are directly adsorbed onto the substrate. We suggest that for these polymers, while the shorter arms flanking the backbones gradually return to the height of the backbones' plane (possibly so as to interact with the Fluorine atoms of the tetrafluorobenzene rings), the longer arms stretching out in the interbackbone region will remain at a larger distance from the substrate. We expect this to be partially due to the attractive lateral van der Waals interaction with the longer arms of neighbouring polymers, and partially due to the steric hindrance with the shorter arms already occupying the lower position. In defective areas, where no energetic advantage can be gained from a regular assembly, the lack of lateral mutual interaction among side chains will force them directly onto the substrate, therefore explaining the dimmer appearance in the STM images.

4. NMR analysis details

Homocoupling (hc) densities and DP_n values were evaluated based on the assignments reported by Broll *et al*.[7] Assignments are exemplarily shown here for P1 only.

Values of DP_n for P1. The entire backbone intensity was divided by the summed end group intensity, taking care that several protons from one unit were not counted twice. Backbone intensity was calculated from the region 9.35 ppm – 8.75 ppm (2.15 protons). End group intensity was calculated from signals 7 (0.15 protons), 10 (0.05 protons) and methyl end group signal intensities 4 in region 7.48 ppm – 7.33 ppm (0.03 protons). Thus, DP_n of P1 was estimated as 2.15/0.23= 9.3 ± 1.0. Signal 3 was neglected due to its low intensity and broad nature. The error was estimated from different variations within this integration procedure, including usage of local baselines, signal 3, different treatments of rather broad and ill-defined methyl group signals, different integration limits and combinations thereof.

Determination of hc values for P1. Generally, the percentage of hc is defined as the number of hc linkages divided by the number of all linkages. Difficulties arose from the overlap of hc signal 1´ with signal group 4. Values of % hc were determined by dividing the hc signal intensity by the backbone intensity, i.e. % DPP hc = %*0.12/2 = 6.0 ± 1.0 %. The error was estimated from using different integration limits.

Values of DP_n for P2. Backbone intensity was calculated in the same way as for P1 (2.06 protons). End group intensity was calculated in the same way as for P1 except that signal 8 (0.07 protons) was used instead of 7 and 3 (0.18 protons) instead of signal 10, giving better reliability. Methyl end group intensity was estimated as 0.025, giving a total end group intensity of 0.275 and a DP_n of 2.06/0.275 = 7.5 ± 3.0. The error was estimated from using different integration limits, whereby the larger value arose from a significantly more noisy baseline as well as unknown signals in the homocoupling region.

Determination of hc values for P2. As can be seen from the limited quality of the spectrum shown in **Figure S8** this estimation was mostly unreliable. Nevertheless the extraction of a hc value was still attempted. Difficulties arose especially from the very broad nature of signal 1´ that appeared in between two other broad signals of unknown origin. Using an intensity of 0.04 for hc signal 1´ furnished % DPP hc = %*0.04/2 = 2 ± 1 %. The error was estimated from using different integration limits and local baselines.

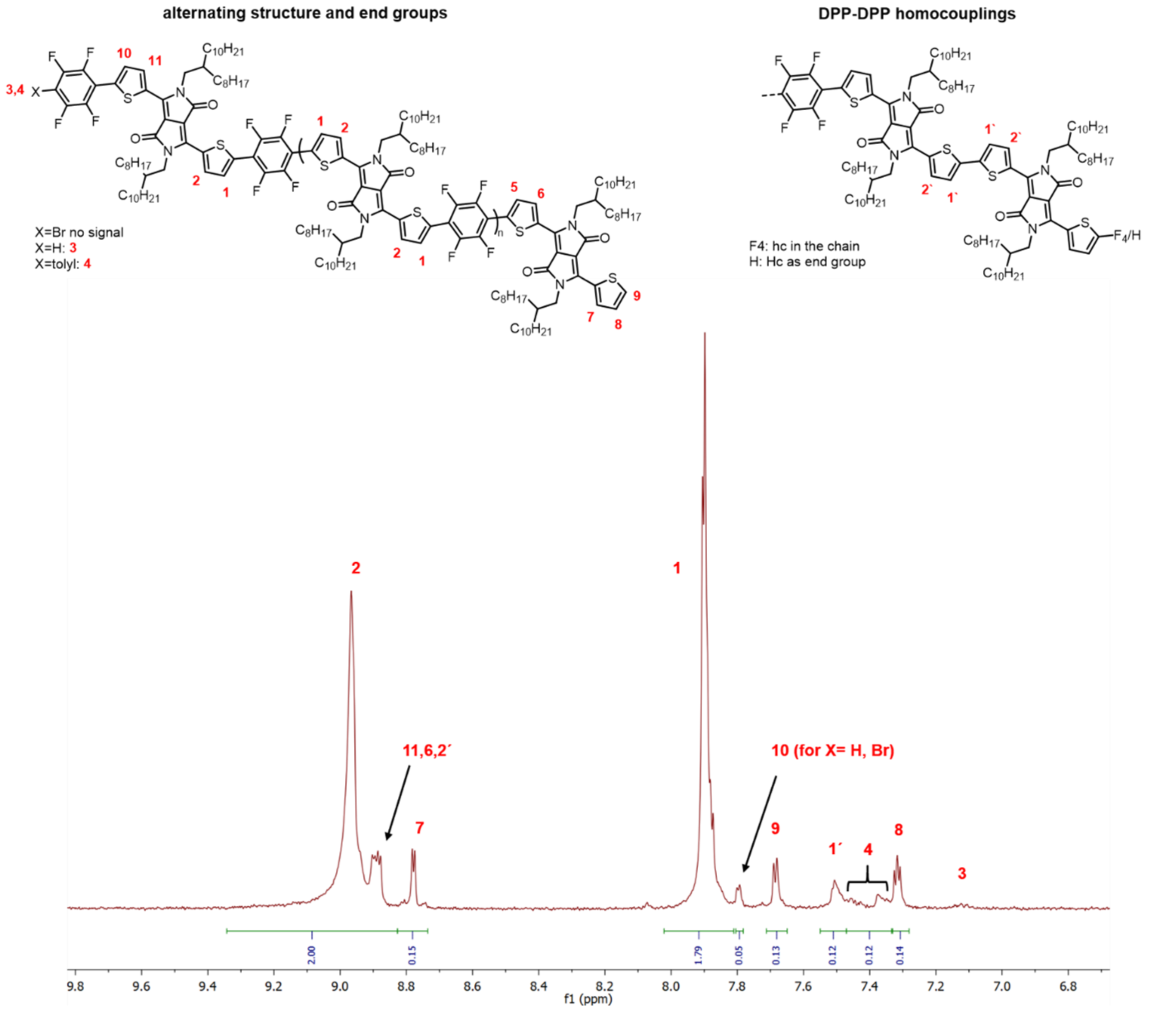

Figure S7. ^{1}H NMR spectrum (aromatic region) of P1 in $C_2D_2Cl_4$ with assignments.

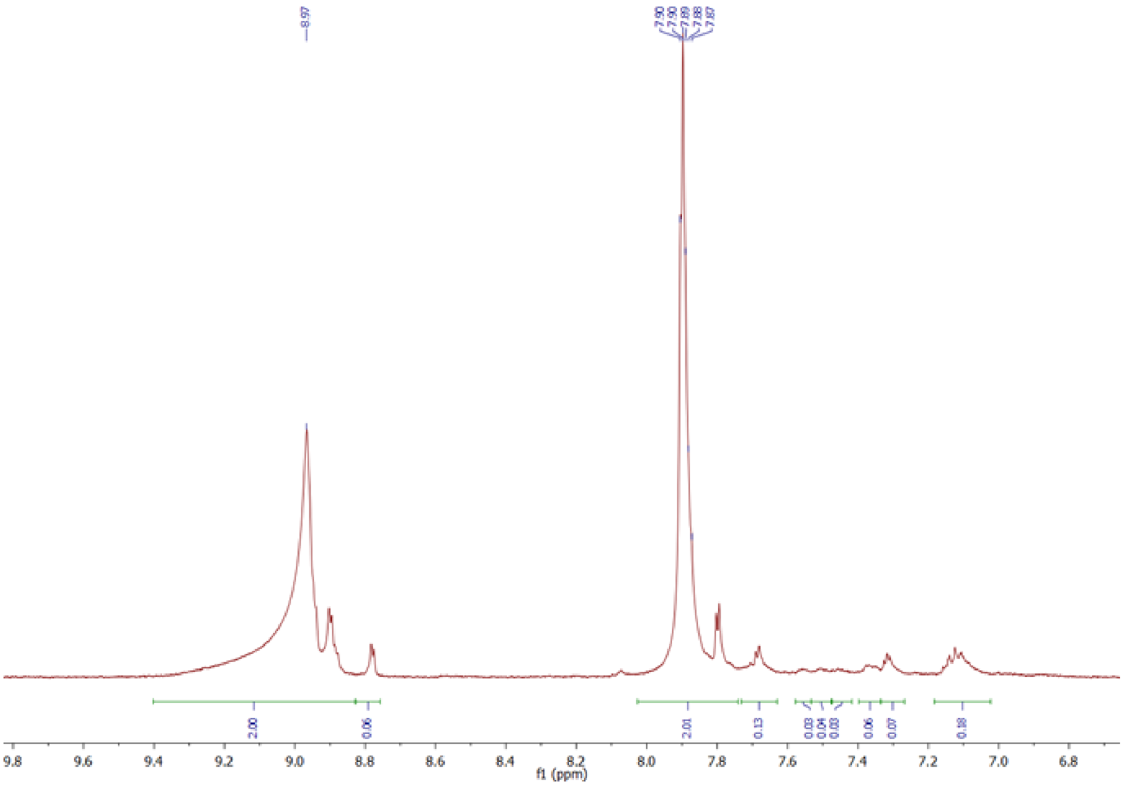

Figure S8. ^{1}H NMR spectrum (aromatic region) of P2 in $C_2D_2Cl_4$.

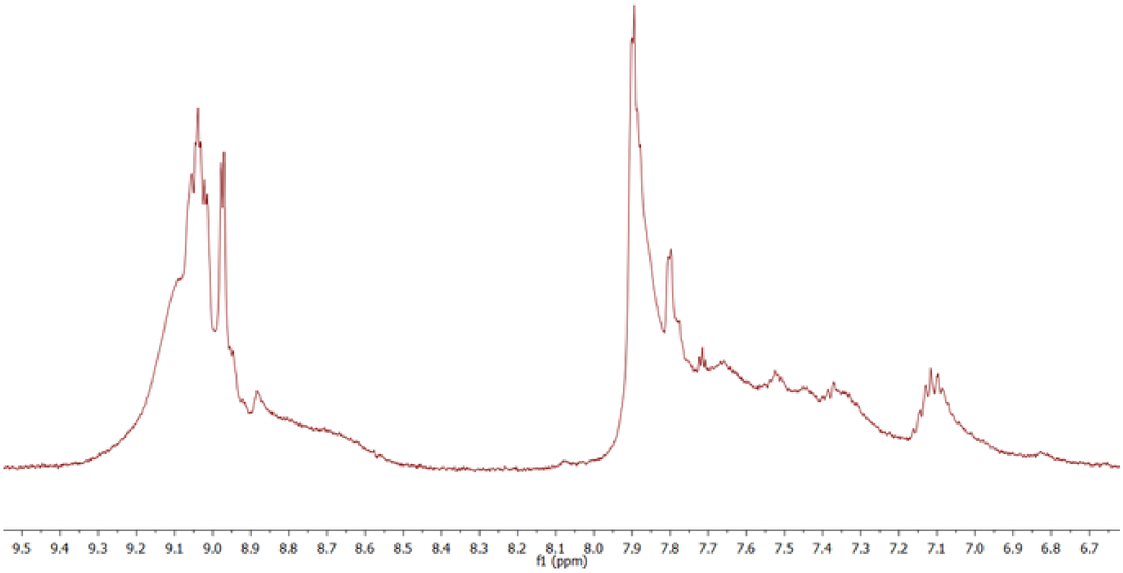

Figure S9. ^{1}H NMR spectrum (aromatic region) of P3 in $C_2D_2Cl_4$.

5. Determining polymer length profiles from STM images

The polymer length distribution data were obtained by directly measuring the length of a large number of individual polymer strands as shown in green in **Fig. S10b**. To avoid systematic errors in the length measurements due to factors related to the scanning procedure (e.g. thermal drift, mechanical piezo creep, miscalibrations), for each analysed image, about 10 well-resolved polymers were chosen, uniformly sampled over the whole imaged area. For these polymers the number of repeat units was directly counted from the image. This number was then compared to the number of repeat units obtained dividing the length of the profile measurements by the length of a single monomer unit (evaluated from molecular models optimized via the MMFF94 force field in the Avogadro molecular editor). In case of mismatch between these values, the required rescaling and/or skewing of the images was applied. Modifying the images was rarely necessary (2 images out of a total of 32). In the case of P1, a further control analysis was performed: the length distributions were evaluated separately for regions of low and high surface density in order to assess any possible role of local polymer coverage on the length distribution of the polymers. No significant variations were found in the two distributions, as they yield the same average mass values within the error bars.

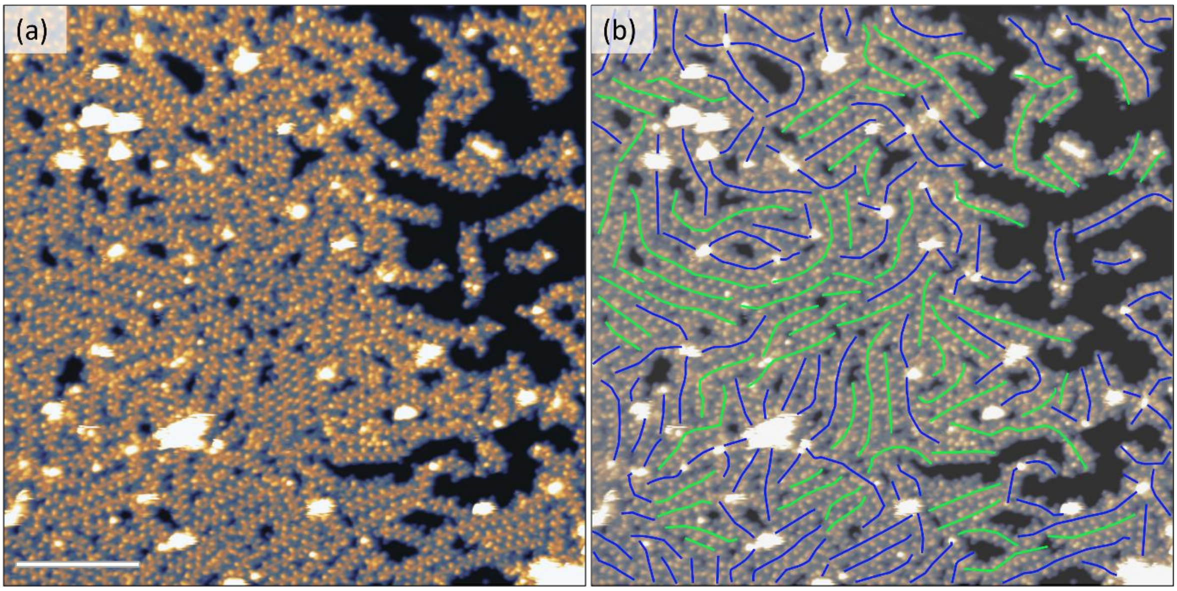

Figure S10. Example of the line-profile process for the acquisition of length distributions of the polymers. An image for P3 is shown here as an example, but the process is identical for P1 and P2. (a) Larger scale STM image. (b) Line-profiles are drawn over the STM image after the calibration process. Profiles corresponding to fully visible polymers entirely contained in the image (green) are recorded separately from profiles of polymers that cannot be fully followed (blue). The two resulting distributions are combined through statistical approaches based on survival analysis (see main paper and section SI6). Scale bar corresponds to 20 nm. The STM image was acquired in constant current mode with tunnelling parameters 1.1 V, 100 pA.

The length of polymers that started and ended within an image were recorded in one list (green profiles in **Fig. S10b**). Another list was used to record polymers that either crossed the borders of the image or crossed each other in regions where they could not be unambiguously distinguished/resolved (blue profiles in **Fig. S10b**). The resulting distributions have been reported in **Fig. 3** and **Fig. S11**, respectively. All images analysed to gather these length distributions had a lateral size of 80 nm. To construct the distributions shown in **Fig. 3**, 606, 613 and 604 complete polymers were counted for P1, P2 and P3 respectively, and 446, 472 and 441 partial polymers were measured for the distributions in **Fig. S11**.

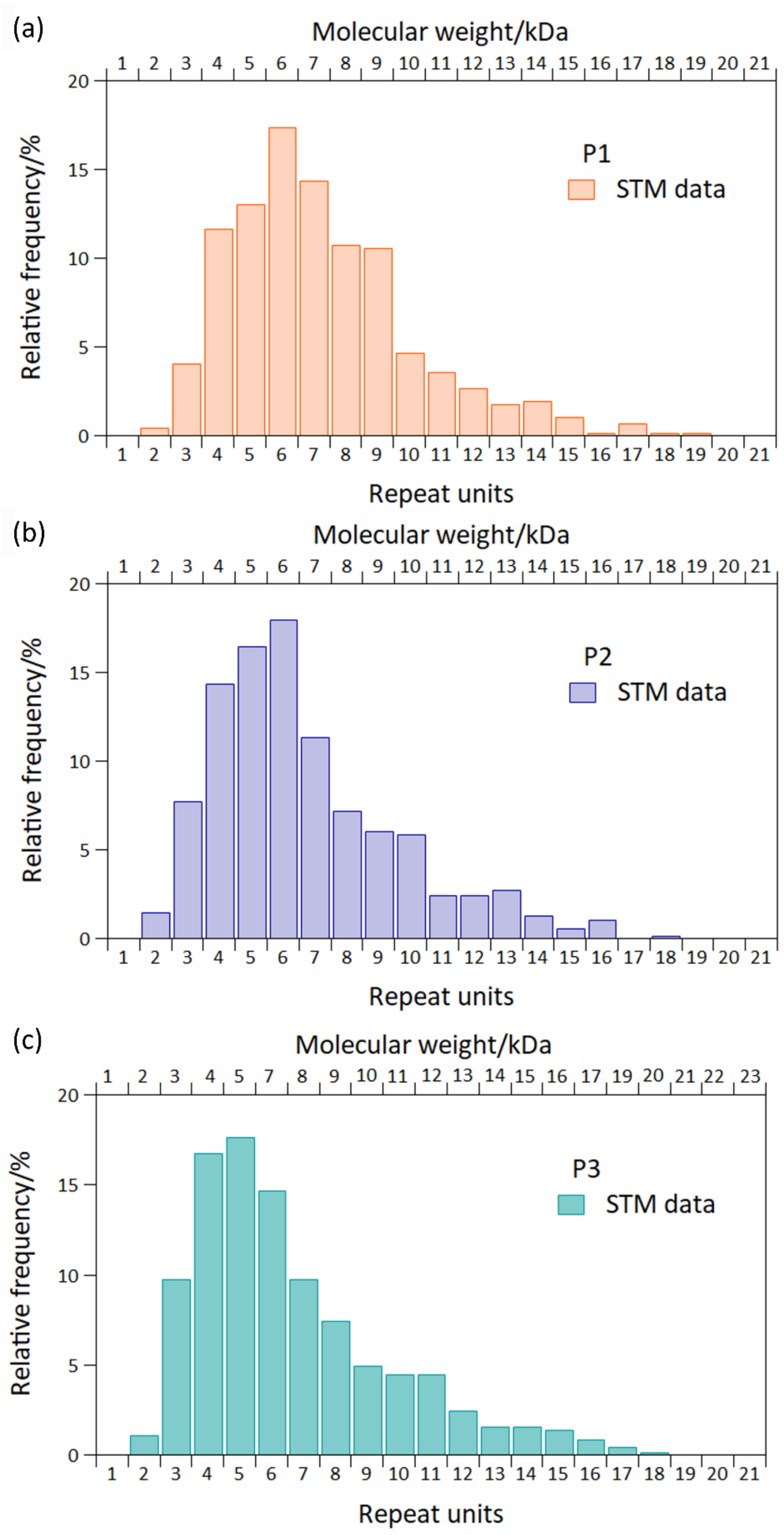

Figure S11. "Longer than" length distributions obtained from measuring a large number of line profiles from the STM images. Only partial polymers that are not fully contained within an image or that are only partially visible are included in these distributions. (a) shows the distribution for P1, (b) for P2 and (c) for P3. These "longer than" distributions were used to correct the length distributions of polymers fully recognisable and contained in the STM images via two statistical approaches from survival analysis.

6. Corrections to length distributions

Since STM images are finite in size, some polymers are not fully comprised within the scanning area as they start/end outside an image. When evaluating the polymer length distribution, ignoring these partially-imaged polymers (blue in **Fig. S10b**) and only considering polymers that are fully included in an STM image (green in **Fig. S10b**) would introduce a systematic error. This would lead to underestimating the length distributions, as longer polymers tend to cross the image borders more than shorter ones. Moreover, discarding the partially imaged polymers would mean discarding almost half of the information contained in an STM image, as can be seen in **Fig. S10b** if the blue and green polymers are compared.

However, one can use the distribution of partially-imaged polymers – which is also evaluated from the STM images, see **Fig. S11** – to correct and complete the data obtained from the "full polymer" distribution. Here we apply, for the first time to the study of conjugated polymers, the statistical tool of survival analysis (which is typically used to model the survival time of patients in medical studies[8]). Survival analysis allows us to evaluate the "correct" mass distribution of conjugated polymers based on STM images. In particular, two approaches have been used in this work, and the results compared. The first one is the Kaplan-Meier estimator, a *non-parametric approach* where no assumptions on the functional shape of the final mass distributions are made. The second approach is a *parametric approach*, where it is assumed that the final mass distributions combine a Flory-Schultz distribution (a geometric distribution)[9] and a low-mass filter (that represents the Soxhlet extraction the polymer is subjected to at the end of the synthesis process In order to eliminate unreacted monomers, residual catalyst and short oligomers, see the polymer synthesis section). By comparing the results from these two approaches, we can assess whether the distributional assumptions in the parametric approach are appropriate.

The mathematical description of these two methodologies is detailed in the following.

<u>Non-parametric approach</u>

As for the parametric approach, the final aim is to estimate the so-called survival function, $S(x)$, *i.e.* the probability that the polymer exceeds length x (*n.b.* x represents the number of polymer repeat units and is thus an integer natural number). Once this is known, the probability of the polymer having exactly length x, $R(x)$, can be obtained as the reduction in the survival curve at each length, *i.e.* the discrete derivative of the survival curve. $R(x)$ can then be used to calculate the required moments of the distribution, which are the quantities typically used in polymer chemistry to characterise the polymer mass distributions. For example, the first moment (or the polymer mean length) is proportional to what in polymer chemistry is called the number average molar mass, M_n, while the second moment corresponds to the mass average molar mass, M_w (see Section 10).

In the non-parametric approach, the Kaplan-Meier estimator was used to estimate $S(x)$. As discussed earlier, by analysing a large number of STM images (referred to as the *sample* in the following), two distributions were evaluated: $d(x)$ (histograms in **Fig. 3**), which counts the number of polymers with exactly length x, and $c(x)$ (histograms in **Fig. S11**), that counts the number of polymers that have been censored at length x, *i.e.* that were observed to have length (at least) x within the STM image but that either crossed the image borders or crossed each other and could thus not be unambiguously distinguished. From these distributions, it is possible to derive a further distribution, $n(x)$, that counts the number of polymers that have length at least x (*i.e.* with length $\geq x$). In fact, by definition,

$$n(x) = \sum_{i \geq x} d(i) + \sum_{i \geq x} c(i)\,. \tag{1}$$

where the index i identifies the (discrete) length of a polymer.

A more convenient relationship between $n(x)$, $d(x)$ and $c(x)$ can be obtained by writing

$$n(x) = \left(d(x) + \sum_{i \geq x+1} d(i) \right) + \left(c(x) + \sum_{i \geq x+1} c(i) \right) = d(x) + c(x) + n(x+1), \tag{2}$$

or

$$n(x+1) = n(x) - d(x) - c(x). \tag{3}$$

Equation (3) can in fact be used iteratively to build $n(x)$ from $d(x)$ and $c(x)$, noting that $n(0)$ is, by definition, the size of the entire sample, *i.e.* the total number of polymers counted.

The next step in the derivation of $S(x)$ is to realise that the distributions $n(x)$ and $d(x)$ can be used to estimate $S(x)$. In order to demonstrate this, let us first express $S(x)$ in an alternative way, by considering the random variable L of the length of a polymer, and the probability of event $L > x$ happening, *i.e.* the probability that a polymer, belonging to the entire polymer population, has length larger than x. By definition,

$$S(x) = \Pr(L > x). \tag{4}$$

Let's now consider the compound probability of events $L > x$ and $L > x - 1$, which is given by

$$\begin{aligned}\Pr(L > x \ \cap L > x - 1) &= \Pr(L > x - 1) \Pr(L > x | L > x - 1) \\ &= \Pr(L > x) \Pr(L > x - 1 | L > x). \end{aligned} \tag{5}$$

Since the conditional probability on the right-hand side of equation (5) is clearly equal to unity (i.e. $\Pr(L > x - 1 | L > x) = 1$), equation (5) can be rewritten as

$$\Pr(L > x - 1) \Pr(L > x | L > x - 1) = \Pr(L > x) \tag{6}$$

or, recalling the definition in equation (4),

$$S(x-1) \Pr(L > x | L > x - 1) = S(x). \tag{7}$$

In order to express the conditional probability in equation (7) as a function of the distributions $n(x)$ and $d(x)$, we first note that the three events $L > x$, $L = x$ and $L < x$ are mutually exclusive and exhaustive and that, therefore,

$$\Pr(L > x \cup L = x \cup L < x) = 1. \tag{9}$$

This equality remains true even if the combined event is conditioned to any other event; thus,

$$\Pr(L > x \cup L = x \cup L < x | L > x - 1) = 1. \tag{10}$$

Because the three events are mutually exclusive

$$\Pr(L > x \cup L = x \cup L < x | L > x - 1) = \Pr(L > x | L > x - 1) + \Pr(L = x | L > x - 1)$$

$$+\Pr(L < x | L > x - 1) = 1. \quad (11)$$

The last probability on the right-hand side of equation (11) is null and thus equation (11) can be rewritten as

$$\Pr(L > x | L > x - 1) = 1 - \Pr(L = x | L > x - 1). \quad (12)$$

If the sample of analysed polymers is large enough to represent well the entire polymer population, the conditional probability on the right-hand side of equation (12) can be estimated as the number of polymers counted to have length exactly equal to x, *i.e.* $d(x)$, divided by the number of polymers that have length $\geq x$, *i.e.* $n(x)$, or

$$\Pr(L = x | L > x - 1) = \frac{d(x)}{n(x)} . \quad (13)$$

Thus, equation (12) becomes

$$\Pr(L > x | L > x - 1) = 1 - \frac{d(x)}{n(x)} \quad (14)$$

and, as a consequence, equation (7) can lastly be rewritten as

$$S(x) = S(x - 1)\left(1 - \frac{d(x)}{n(x)}\right). \quad (15)$$

Noting that, by definition, $S(0) = 1$, equation (15) can be applied recursively to arrive to the final equation

$$S(x) = \prod_{i<x}\left(1 - \frac{d(i)}{n(i)}\right), \quad (16)$$

which is the Kaplan-Meier estimator for the probability that a polymer is longer than x.

The calculation of equation (10) was implemented in the statistical programming language R using the “survival” package.[10]

Parametric approach

Using a parametric approach allows the available data to inform the shape of the whole distribution, so we can estimate the proportion of polymers with lengths that do not appear in the dataset. As stated in the main text, in the parametric approach we describe the relationship between polymer lengths and their observed frequencies through a Flory-Schultz geometric distribution, which is the length distribution expected for an ideal step-growth polymerisation process.[9] To fully describe the experimental materials, though, it is necessary to combine this function with a filtering function representing the purification steps (Soxhlet extraction) performed on the polymers to remove shorter oligomers, unreacted monomers and residual catalyst. A logistic function was chosen to describe this low mass filtering.

The resulting probability function for the length of the polymer L is thus of the form

$$\Pr(L = x|p, k, x_0) = C \frac{1}{1 + e^{-k(x-x_0)}} (1-p)(p)^{x-1}, \quad (17)$$

where C is the normalising constant, L is the random variable representing the length of the polymer, x_0 is the centre of the logistic curve, k is its steepness, and p is the parameter from the geometric distribution (the *extent of reaction* in the Flory–Schulz distribution).

The survival function, $S(x) = \Pr(L > x)$, can be easily calculated from the probability function (17) by considering that

$$\Pr(L > x) = 1 - \Pr(L \leq x) \quad (18)$$

and by evaluating the probability on the right-hand side of equation (18) as

$$\Pr(L \leq x) = \sum_{i=1}^{x} \Pr(L = i). \quad (19)$$

Thus, if the probabilities are obtained from the parametric functional dependence in equation (17), the survival function can be expressed as

$$S(x|p, k, x_0) = 1 - \sum_{i=1}^{x} C \frac{1}{1 + e^{-k(i-x_0)}} (1-p)(p)^{i-1}. \quad (20)$$

The method of *maximum likelihood estimation* was used to find the best fit parameters for the probability distribution (17). To this aim, a likelihood function needs to be built that considers both type of observations that were done experimentally, *i.e.* measurements of the full length of polymers and the censored measurements. Given the measurement of a full polymer length x_1, the associated likelihood is equal to the probability $\Pr(L = x_1|p, k, x_0)$, i.e., via equation (17), to

$$\Pr(L = x_1|p, k, x_0) = C \frac{1}{1 + e^{-k(x_1-x_0)}} (1-p)(p)^{x_1-1}. \quad (21)$$

Similarly, given the measurement of a censored polymer of length at least y_1, the associated likelihood is equal to the probability

$$\Pr(L \geq y_1|p, k, x_0) = \Pr(L > y_1 - 1|p, k, x_0), \quad (22)$$

where we have used the fact that the lengths are measured as multiples of the polymer repeat units and are thus represented by integer numbers. Recalling the definition (4) of the survival function and expression (20), the likelihood (22) for the measurement of a censored polymer of length at least y_1 becomes

$$S(y_1 - 1|p, k, x_0) = 1 - \sum_{i=1}^{y_1-1} C \frac{1}{1 + e^{-k(i-x_0)}} (1-p)(p)^{i}. \quad (23)$$

The likelihood $\mathcal{L}$ associated with n observed full polymer lengths $(x_1, x_2, \cdots, x_n)$ and m observed censored polymer lengths $(y_1, y_2, \cdots, y_m)$ is simply the product of the likelihoods associated to the individual observations, assuming that the events are independent. Thus,

$$\mathcal{L}(p,k,x_0) = \prod_{i=1}^{n} \Pr(L = x_i|p,k,x_0) \prod_{j=1}^{m} S\big(y_j - 1\big|p,k,x_0\big) \tag{24}$$

with the explicit dependence on the parameters p, k and x_0 being given through equations (17) and (20). Finally, maximum likelihood estimates of the parameters p, k and x_0 (i.e. the parameter values that were the most likely to produce the entire set of experimental data) are obtained by numerical maximisation of $\mathcal{L}$ (in reality, in order to provide numerical stability, what is maximised is the log-likelihood function, *i.e.* the logarithm of equation (24)).

The normalising constant C was calculated by summing over the first 200 terms of the unnormalised probability distribution. The maximum likelihood estimates of the parameters p, k and x_0 were obtained using the function “optim” in the statistical programming language R.[11] The corresponding marginal confidence intervals were calculated from the inverse of the numerical hessian matrix returned by “optim”.

The results for the two survival analysis approaches are shown in **Fig. S12**.

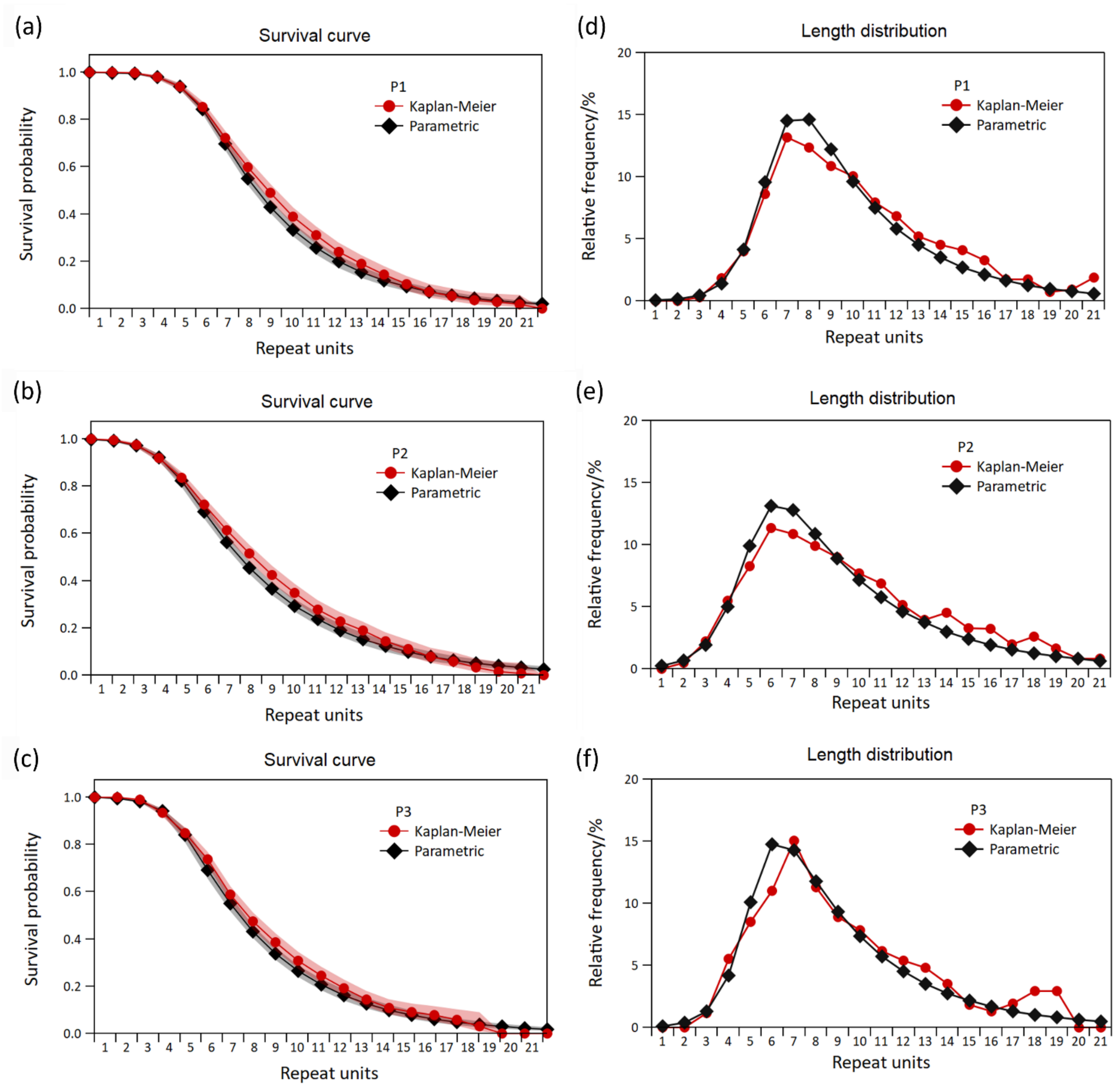

Figure S12. Left column, plots of the survival function $S(x)$ obtained through the two survival analysis approaches used in this work: Kaplan-Meier non-parametric approach (red circles) and parametric approach (black diamonds) for (a) P1, (b) P2 and (c) P3 respectively. The corresponding 95% confidence intervals are shown as semi-transparent thick lines with the appropriate colour. Right column, corresponding length distributions for (d) P1, (e) P2 and (f) P3, respectively.

7. Effective extents of reaction

Having access to the full mass distributions allows one to use the STM data to further characterise conjugated polymers in ways that are inaccessible by NMR. In fact, as shown in Section 6, the mass distributions can be fitted by the product of a logistic curve and a Flory-Schultz function, as expressed by equation (17). As a consequence, three parameters can be extracted, k, x_0, and p, respectively. k and x_0 represent the steepness and the centre of the logistic curve, respectively, and thus measure the low-mass filtering effect of the Soxhlet extraction. On the other hand, p, the so-called fractional monomer conversion or extent of reaction, is intrinsic to the polymerisation process. This parameter is related to the fraction of unreacted monomers remaining after the polymerisation reaction has terminated and describes the probability of an additional polymerisation step to occur, thereby giving an indication on the overall favourableness of the polymerisation process.[9]

Theoretically, the best way to evaluate p would be to analyse the raw, unfractionated solution containing all reactants and reaction products. From a practical point of view, however, this is complicated by the challenge of obtaining an entirely unfractionated solution which is compounded by the experimental intricacies of filtering organics over silica to remove catalyst and base without loss of aggregates (larger chains), thereby aiming for a clear separation between organics and inorganics. If the low-mass filtering operated by the Soxhlet extraction was fully described by a simple logistic function, equation (17) would completely capture its effect and the p value obtained from fitting a fractioned sample would be a perfect representation of the extent of reaction. However, the effect of the Soxhlet extraction is often more complex and there is evidence, in particular for DPP-based polymers, that the removal of low-mass species can be incomplete and not just described by a simple logistic function. As such, the p value obtained from fitting a fractioned sample should not be considered as being a direct representation of the extent of reaction, although it is surely related to this quantity. However, this effective p parameter can still be used as a precise and detailed parameter to characterise the mass distribution of a fractioned sample which, as shown in Section 6, follows very closely the functional dependence expressed by equation (17).

In particular, when applied to the chloroform fractions of polymers P1, P2 and P3, the fitting procedure results in effective p values equal to 0.77 ± 0.02, 0.80 ± 0.02**,** 0.78 ± 0.02, respectively. The fact that these are quite similar to each other demonstrates that, in this case, the polymerisation conversion is not significantly influenced neither by the change in polymerisation conditions between P1 and P2 nor by the use of heavier side chains in P3.

8. SEC analysis details

The SEC mass distributions are shown in **Fig. S13a**, normalised in intensity to 1 for comparison purposes. All distributions are characterised by two main peaks, with the one at higher mass typically being associated with aggregation of the polymers. While for P1 and P2 the aggregation shoulder is smaller than the main peak, in the case of P3 the aggregation peak is the dominant one. This agrees with the longer and more flexible side chains of P3 favouring and strengthening lateral interactions between polymer strands.

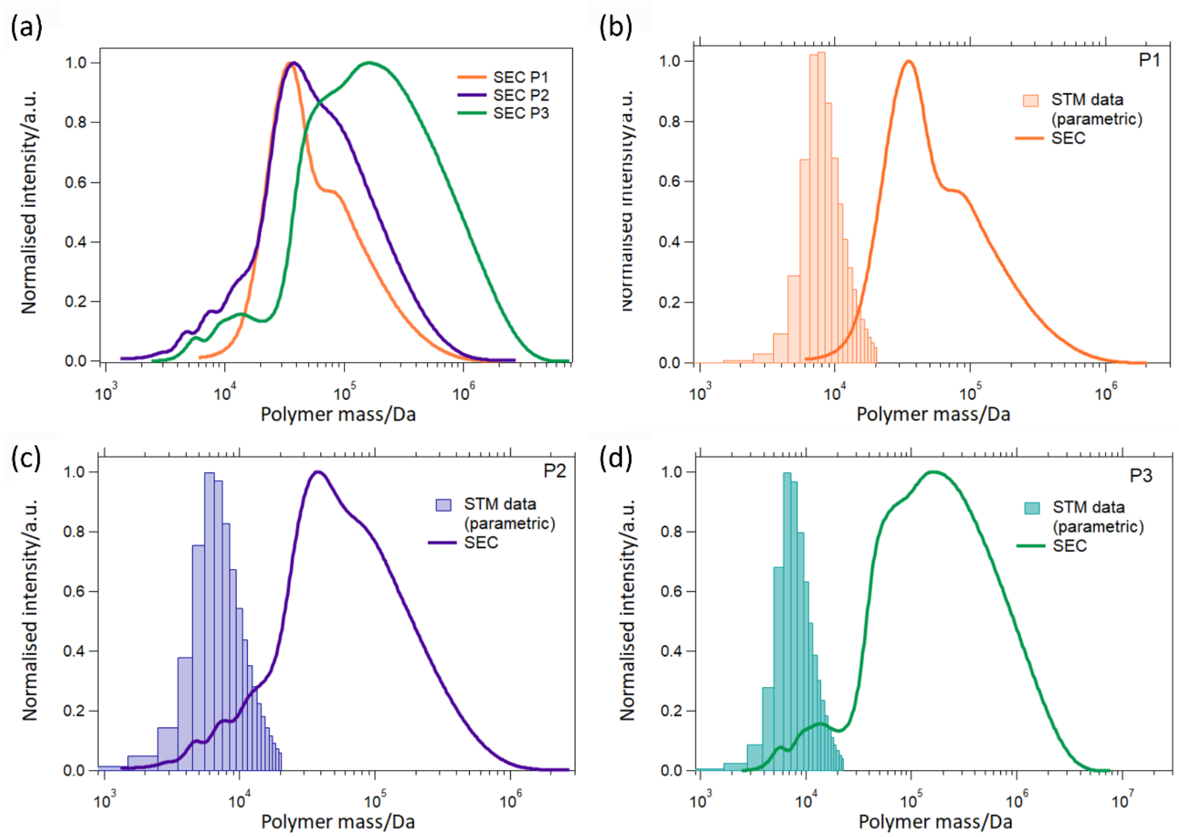

Figure S13. Comparison between SEC and STM mass distributions. (a) SEC mass distributions for the three polymers, showing a similar average mass for P1 and P2 with aggregation shoulders in similar positions, while P3 polymers display a much more extended aggregation shoulder, in agreement with its longer and more flexible side chains promoting intermolecular interactions. Comparisons between the SEC distributions (continuous lines) and the STM histograms (presented in Fig. 3 of the main paper) are shown in panels (b), (c) and (d) for P1, P2 and P3, respectively. The well-known mass overestimation issues of SEC for conjugated polymers are strongly evident.

Using the low-mass peaks present in the SEC mass distributions of P2 and P3, it is possible to recalibrate the mass scale of the SEC spectra. In fact, as discussed in the main paper, a careful look at the SEC curves (**Fig. 4a**) reveals that oligomer peaks, which were probably not completely removed during Soxhlet extraction, are visible in the low-mass regions of P2 and P3. These peaks can be used as a reference to evaluate the functional relationship between the SEC measured masses (determined though universal calibration using the viscometer detector) and their actual values.

In **Fig. S14** each data point represents the mass value at which the maximum intensity of the low-mass peaks in the SEC spectra is measured and is displayed as a function of the actual mass of the corresponding oligomer. In doing so, we assumed that the oligomers: i. are defect-free; ii. have an $(AB)_n$ structure; iii. are hydrogen terminated. This results in an expected mass for the n-oligomer

$$m_n = 2 + n \cdot m_0 \tag{25}$$

where the initial 2 is because of the H-termination of both ends of the oligomer and m_0 is the mass for the AB radical, i.e. the polymer repeat unit. These latter are equivalent to 1007.46 for P1 and P2 and to 1119.67 for P3, respectively (masses in equation (25) are expressed in amu). A degree 2 polynomial function in the form

$$m_{n,\mathrm{SEC}} = a(m_n)^2 + bm_n + c \tag{26}$$

where $m_{n,\mathrm{SEC}}$ is the SEC-measured mass of the n-oligomer and m_n is given by equation (25), is the best fit to the data for both P2 and P3 (see **Fig. S14**), though the parameters are different for the two polymers (see **Table S2**). The inverse functions of these fits give the scaling factors that were used to recalibrate the horizontal SEC mass axis as shown in **Figs. 4b** and **4c** for P2 and P3, respectively.

Table S2. Parameters of the degree 2 polynomials that best fit the relationship between the SEC-evaluated mass of small oligomers of P2 and P3 and their actual masses (see **Fig. S14**).

**polymer**	**a / amu^{-1}**	**b**	**c / amu**
**P2**	$(1.08 \pm 0.08) \times 10^{-3}$	-1.5 ± 0.7	$(3 \pm 1) \times 10^3$
**P3**	$(3.3 \pm 0.4) \times 10^{-3}$	-13 ± 4	$(1.8 \pm 0.8) \times 10^4$

We notice that while the assumption of considering the oligomers as defect-free is surely a good approximation because of the relatively low absolute values of the measured defects content (see **Table 2** in the main paper), we cannot be sure *a priori* about the precise (average) chemical structure of the oligomers. In order to check the effect of different possible end groups on the fitting procedure, we have re-evaluated the best fits to equation (26) where, instead of considering H-(DPP-F4)$_n$-H oligomers, we considered H-(DPP-F4)$_n$-Br and H-(DPP-F4)$_n$-tolyl oligomers. The a, b and c parameters of the corresponding degree 2 polynomials were compatible with those in **Table S2**, within the reported uncertainties. The same is true also when considering H-(DPP-F4)$_{n-1}$DPP-H oligomers.

Finally, it should be considered that in performing this rescaling operation, it has been implicitly assumed that the same functional dependence of the overestimation of the SEC polymer masses determined for short oligomers (< 1.5×10^5 Da, as measured by SEC) holds also for longer polymer chains (> 10^6 Da, as measured by SEC). While it might be possible that this overestimation increases more slowly (or potentially even saturates) for higher masses, there is no experimental evidence to support such an assumption. In fact, at higher masses the SEC measurements are plagued by the other problem of polymer aggregation. Thus, even by directly comparing the SEC-determined masses with the ESD-STM-determined masses, there is no way to disentangle the two effects (**Figs. 4b** and **4c**). As a consequence, the best option is to consider the simplest approximation that the same functional expression of the SEC mass overestimation determined for low masses holds also for higher masses.

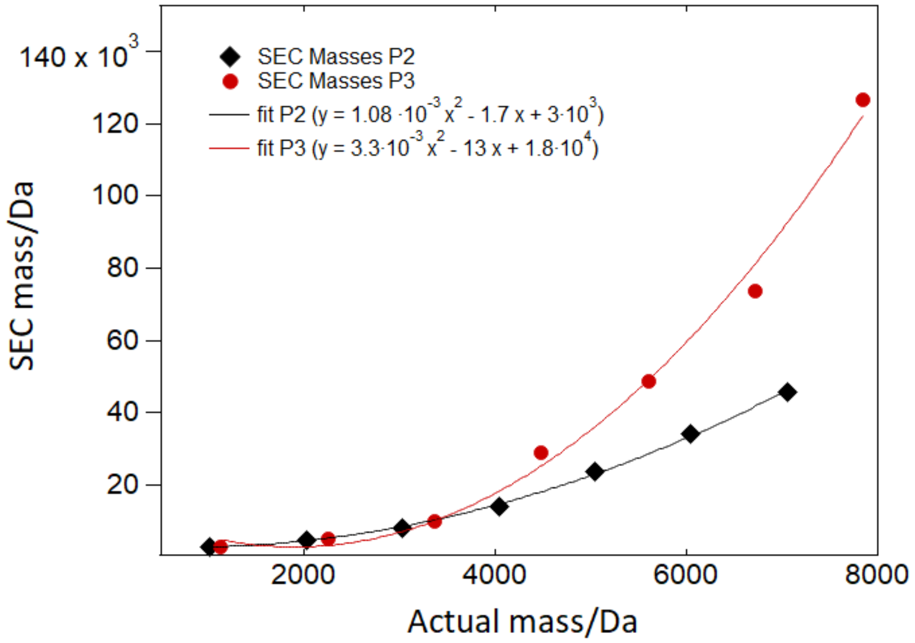

Figure S14. Relationship between the SEC-evaluated mass of small oligomers of P2 and P3 and their actual masses. Black diamonds identify the data points for P2, red circles for P3. The continuous lines represent the corresponding degree 2 polynomial curves that best fit the data. The fit parameters are shown in the legend for P2 and P3, respectively.

9. UV-vis spectroscopy

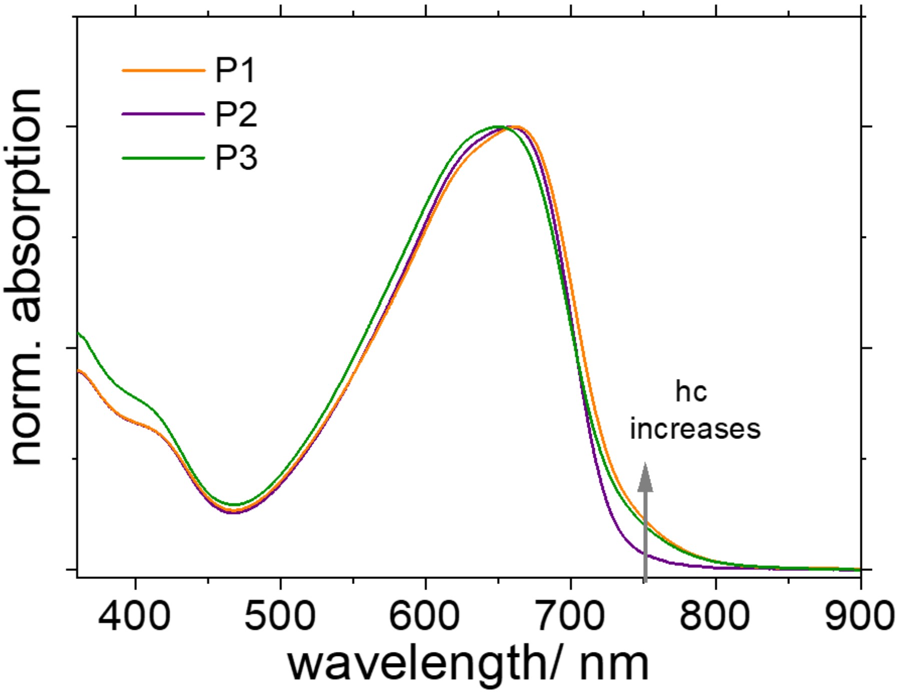

Figure S15. High temperature UV-vis spectroscopy of samples P1-3 in chloronaphthalene at 150 °C. The shoulder at 750 nm arises from hc and is largest for samples P1 and P3 which have the higher hc density.

10. Calculating average values from distributions

Both ESD-STM and SEC provide full polymer mass distributions from which the number average molecular mass, M_n, and the weight average molecular mass, M_w, can be calculated. These do in fact correspond to the first and second moment of the distribution, respectively, and are obtained through the following equations

$$M_\mathrm{n} = \frac{\Sigma M_i N_i}{\Sigma N_i} \tag{25}$$

$$M_\mathrm{w} = \frac{\Sigma M_i^2 N_i}{\Sigma M_i N_i} \tag{26}$$

where N_i is the number frequency of polymer chains of mass M_i. The degree of polymerisation, $\mathrm{DP_n}$, gives the average number of repeat units in a polymer molecule, and can be derived as

$$\mathrm{DP_n} = \frac{M_\mathrm{n}}{M_0}, \tag{27}$$

where M_0 is the molecular weight of the polymer repeat unit.

References

1. Huo, L. *et al.* Bandgap and molecular level control of the low-bandgap polymers based on 3,6-dithiophen-2-yl-2,5-dihydropyrrolo[3,4-c]pyrrole-1,4-dione toward highly efficient polymer solar cells. *Macromolecules* **42**, 6564–6571 (2009).
2. Hanwell, M. D. *et al.* Avogadro: An advanced semantic chemical editor, visualization, and analysis platform. *J. Cheminform.* **4**, 1–17 (2012).
3. Perdigão, L.M.A. LMAPper – Where scanning probe microscopy and molecular visualisation meet. https://sourceforge.net/projects/spm-and-mol-viewer/ accessed on 22.03.2024.
4. Wang, Q. *et al.* Hydrogen Bonds Control Single-Chain Conformation, Crystallinity, and Electron Transport in Isoelectronic Diketopyrrolopyrrole Copolymers. *Chem. Mater.* **33**, 2635–2645 (2021).
5. Moro, S. *et al.* The Effect of Glycol Side Chains on the Assembly and Microstructure of Conjugated Polymers. *ACS Nano* **16**, 21303−21314 (2022).
6. Warr, D. A. *et al.* Sequencing conjugated polymers by eye. *Sci. Adv.* **4**, 0–6 (2018).
7. Broll, S. *et al.* Defect Analysis of High Electron Mobility Diketopyrrolopyrrole Copolymers Made by Direct Arylation Polycondensation. *Macromolecules* **48**, 7481–7488 (2015).
8. Cox, D. R. & Oakes, D. *Analysis of survival data*. *Monographs on Statistics and Applied Probability 21* (CHAPMAN & HALL/CRC, 2018).
9. Odian, G. *Principles of Polymerization*. *The Cambridge Handbook of Stylistics* (John Wiley & Sons, Inc., 2004).
10. Therneau, T. A Package for Survival Analysis in R. R package version 3.1-12. https://cran.r-project.org/web/packages/survival/index.html (2020). Accessed on22.03.2024.
11. R Core Team. A language and environment for statistical computing. *R Foundation for Statistical Computing; Vienna; Austria.* https://www.r-project.org/ (2018). Accessed on 22.03.2024.
